# Supplementary figures and images for: An integrative multitiered computational analysis for better understanding the structure and function of 85 miniproteins
Source: NAR Genom Bioinform. 2025 Dec 3;7(4):lqaf178. doi: 10.1093/nargab/lqaf178 (PMC12673850; doi:10.1093/nargab/lqaf178)

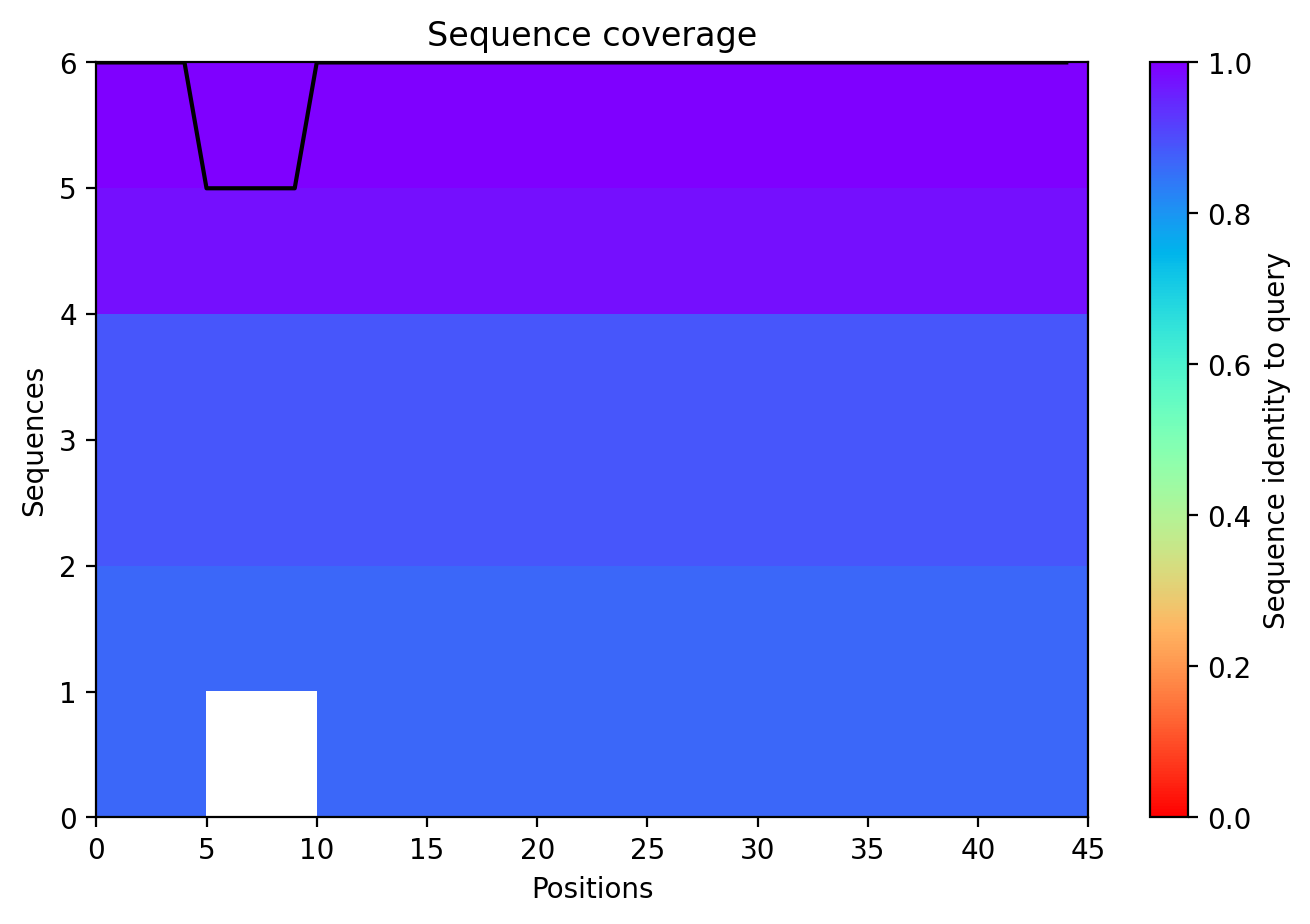

Supplement: lqaf178_Supplemental_Files [file lqaf178_supplemental_files.zip › YX002/msa_coverage.png]

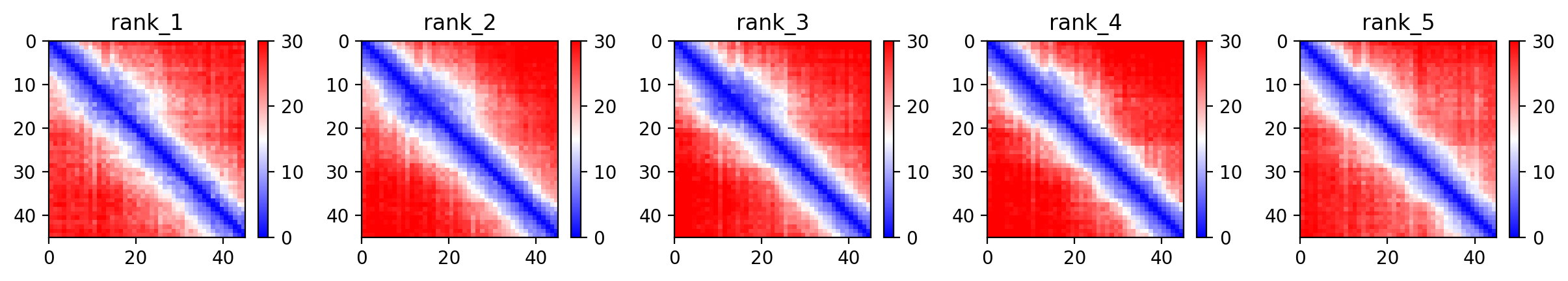

Supplement: lqaf178_Supplemental_Files [file lqaf178_supplemental_files.zip › YX002/predicted_alignment_error.png]

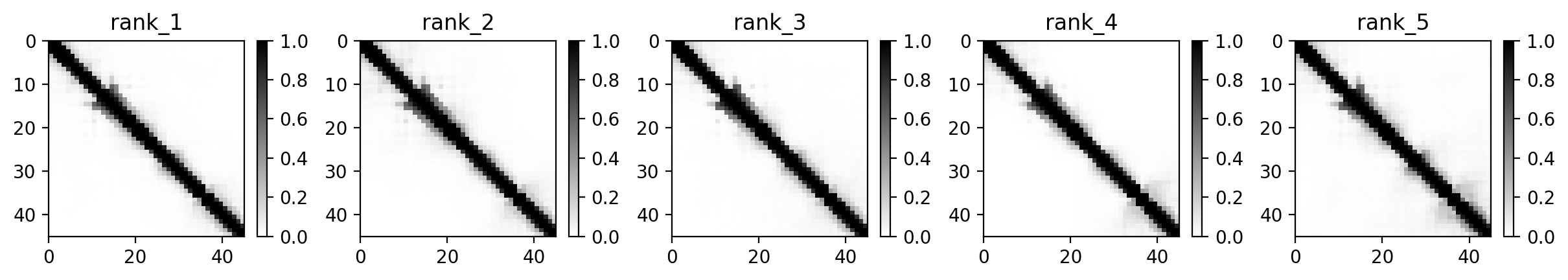

Supplement: lqaf178_Supplemental_Files [file lqaf178_supplemental_files.zip › YX002/predicted_contacts.png]

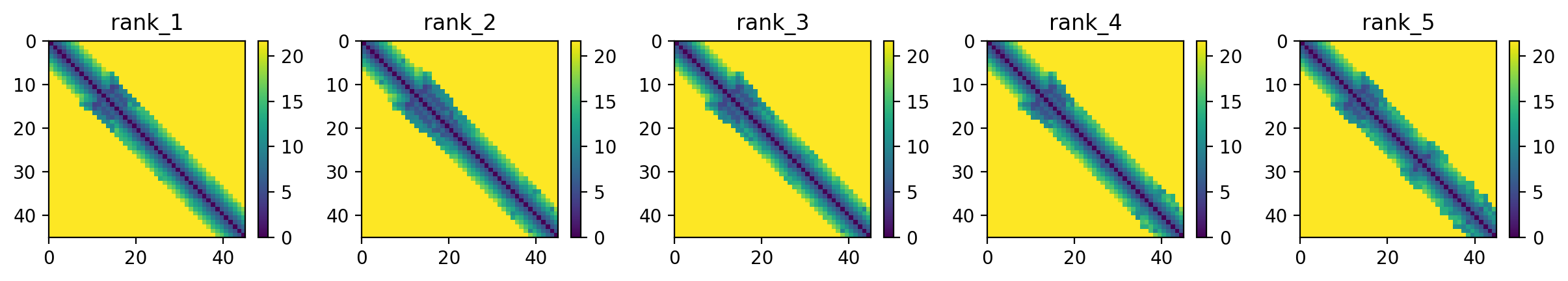

Supplement: lqaf178_Supplemental_Files [file lqaf178_supplemental_files.zip › YX002/predicted_distogram.png]

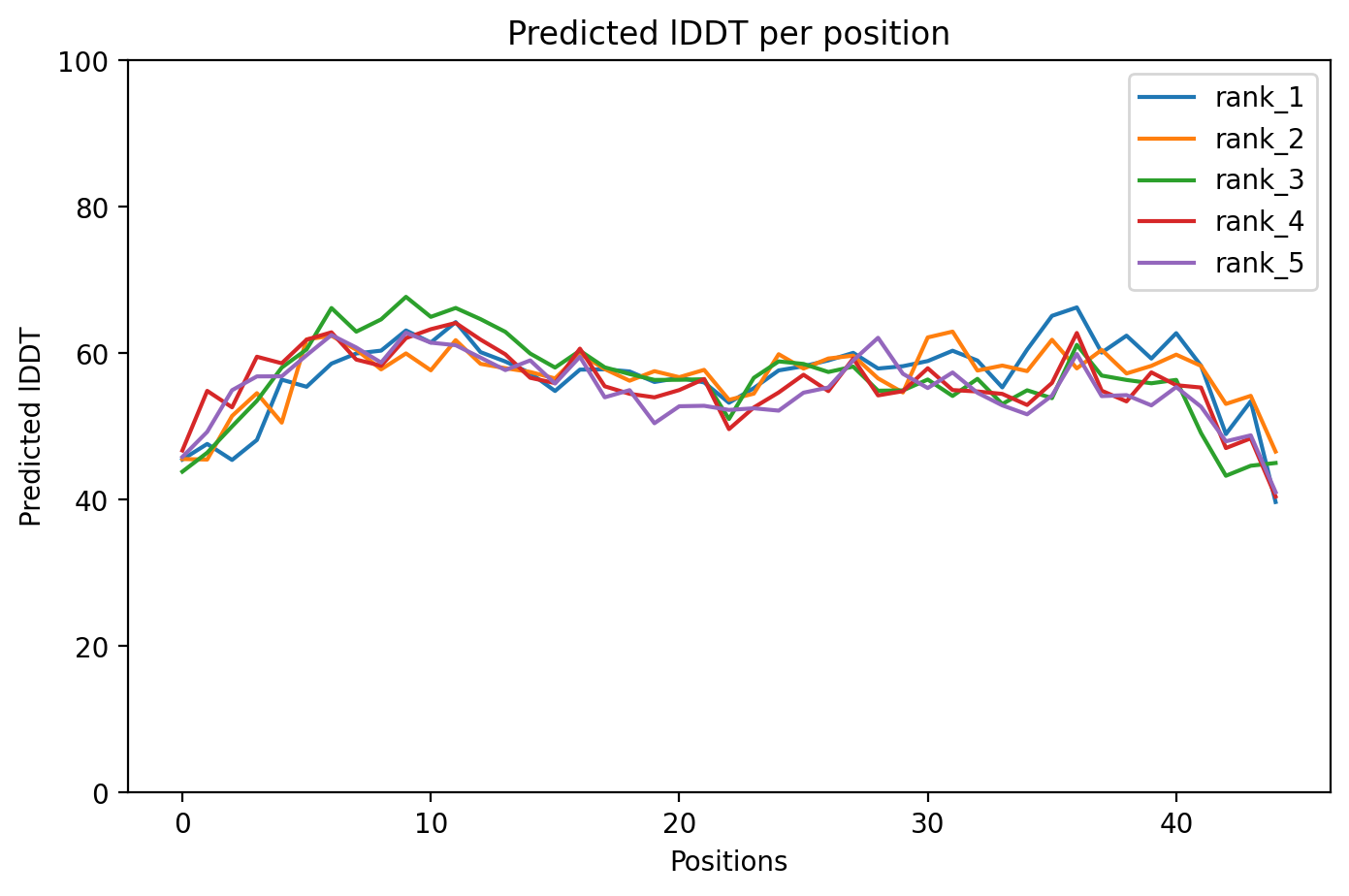

Supplement: lqaf178_Supplemental_Files [file lqaf178_supplemental_files.zip › YX002/predicted_LDDT.png]

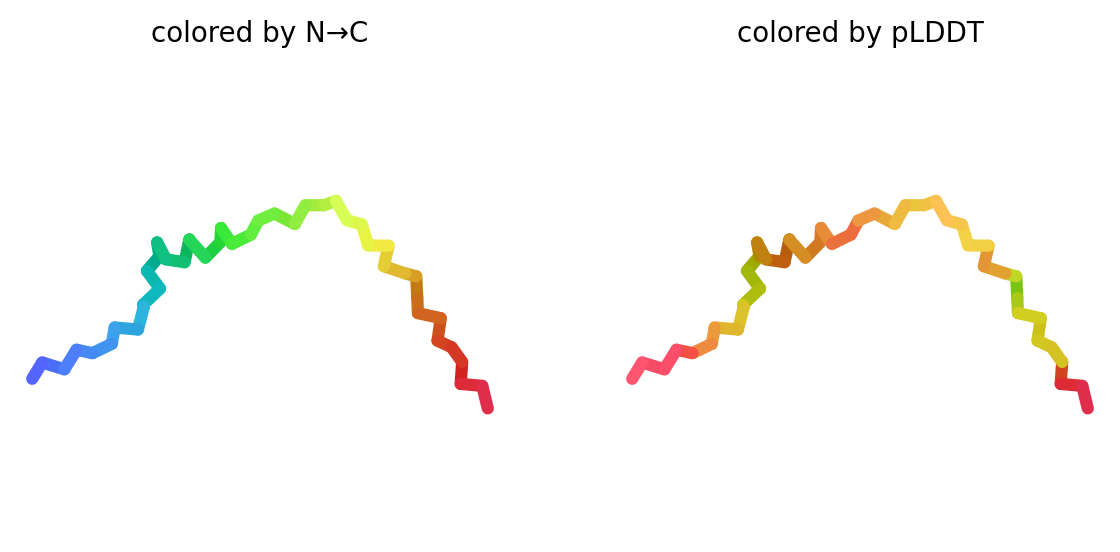

Supplement: lqaf178_Supplemental_Files [file lqaf178_supplemental_files.zip › YX002/rank_1_model_1_ptm_seed_0.png]

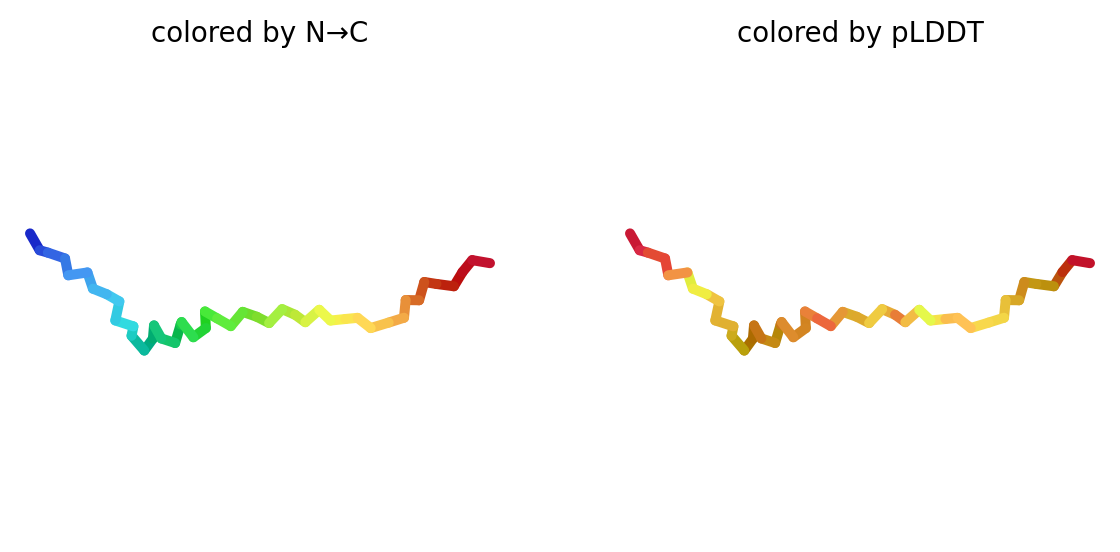

Supplement: lqaf178_Supplemental_Files [file lqaf178_supplemental_files.zip › YX002/rank_2_model_2_ptm_seed_0.png]

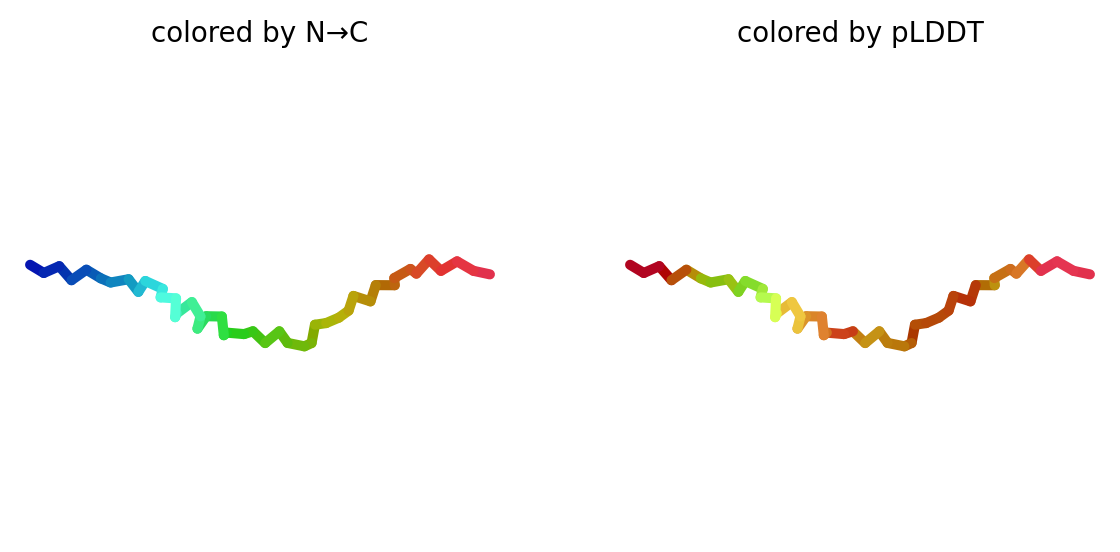

Supplement: lqaf178_Supplemental_Files [file lqaf178_supplemental_files.zip › YX002/rank_3_model_4_ptm_seed_0.png]

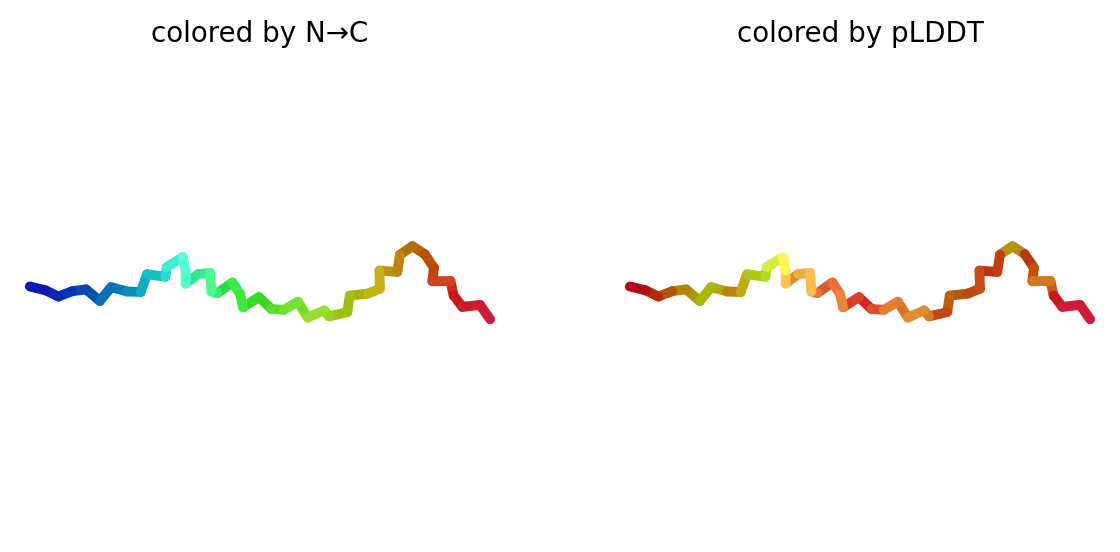

Supplement: lqaf178_Supplemental_Files [file lqaf178_supplemental_files.zip › YX002/rank_4_model_5_ptm_seed_0.png]

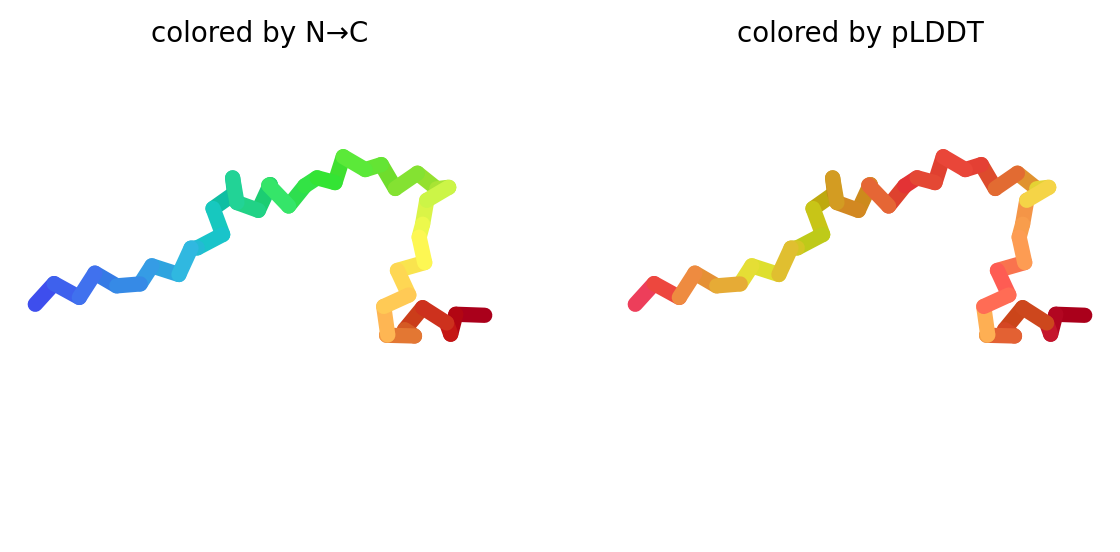

Supplement: lqaf178_Supplemental_Files [file lqaf178_supplemental_files.zip › YX002/rank_5_model_3_ptm_seed_0.png]

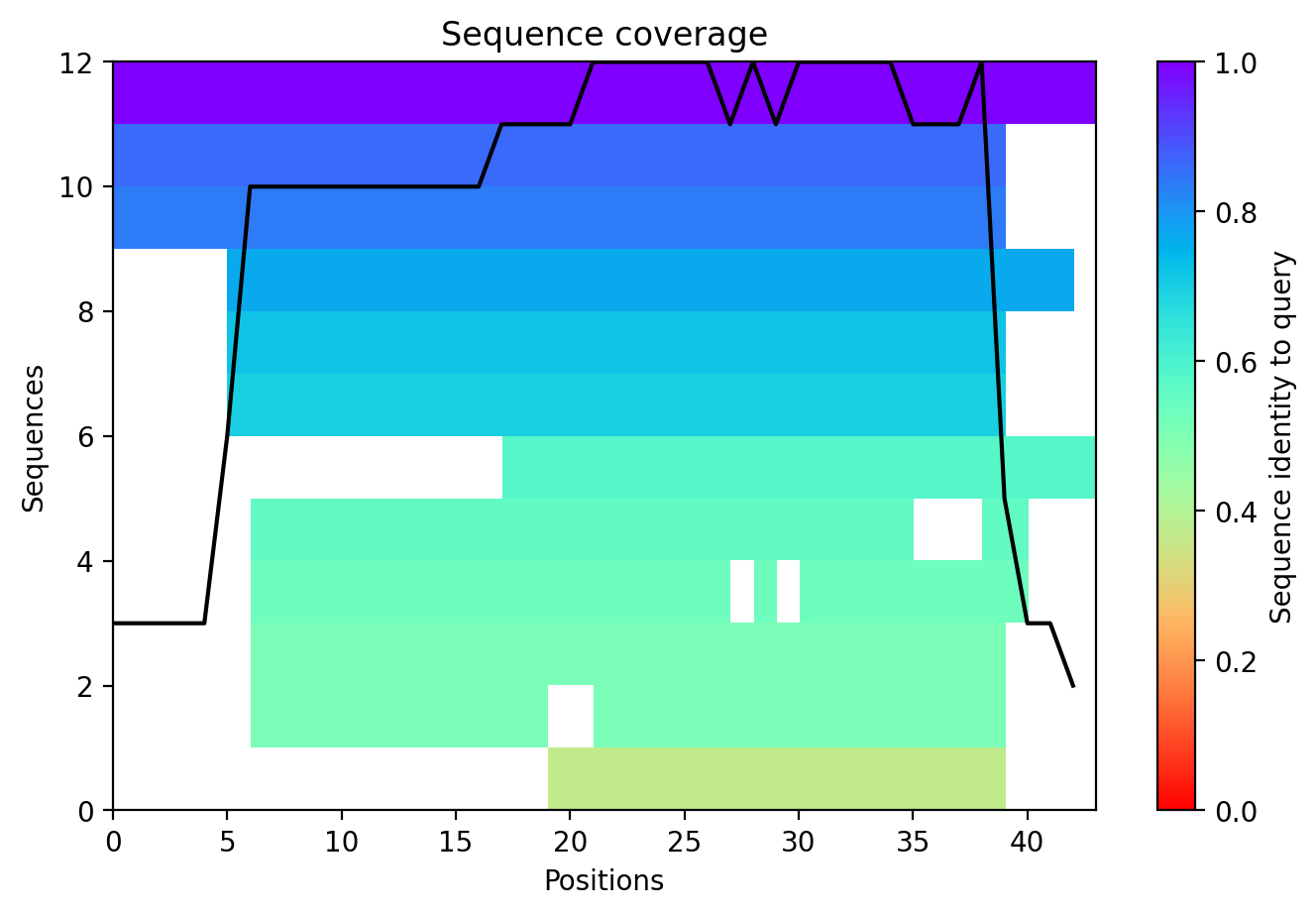

Supplement: lqaf178_Supplemental_Files [file lqaf178_supplemental_files.zip › BAGE1/msa_coverage.png]

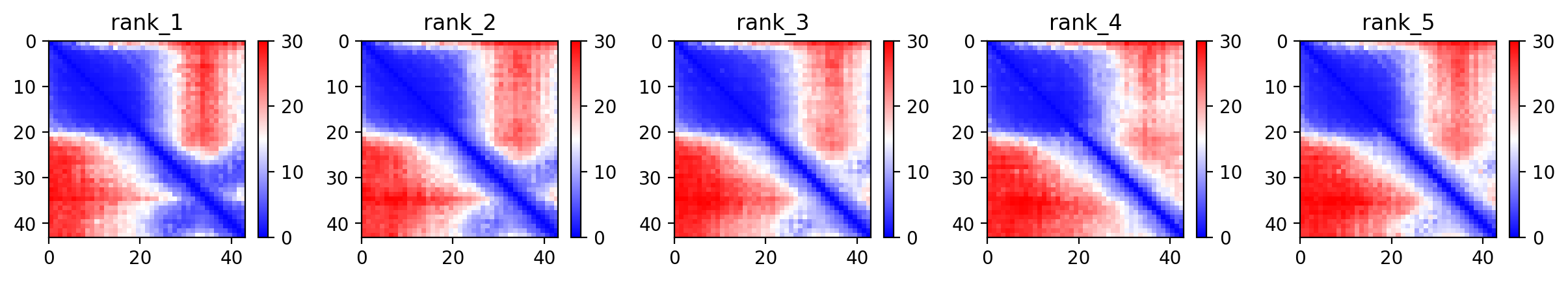

Supplement: lqaf178_Supplemental_Files [file lqaf178_supplemental_files.zip › BAGE1/predicted_alignment_error.png]

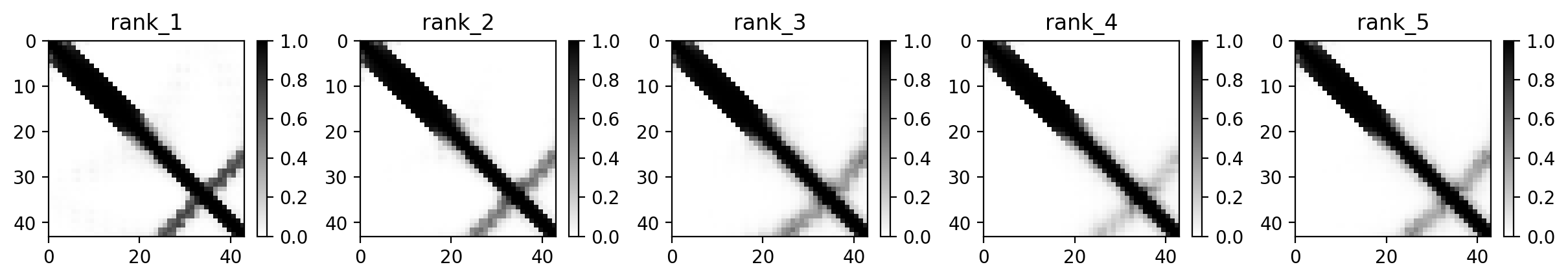

Supplement: lqaf178_Supplemental_Files [file lqaf178_supplemental_files.zip › BAGE1/predicted_contacts.png]

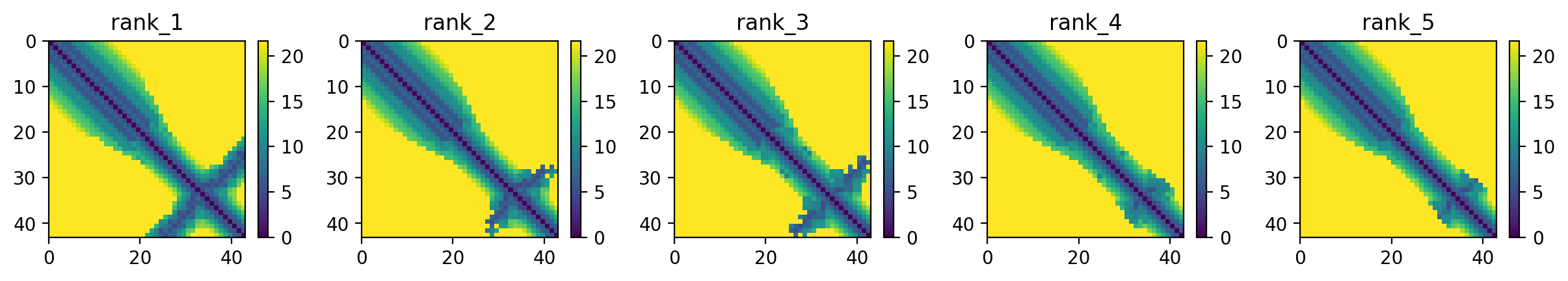

Supplement: lqaf178_Supplemental_Files [file lqaf178_supplemental_files.zip › BAGE1/predicted_distogram.png]

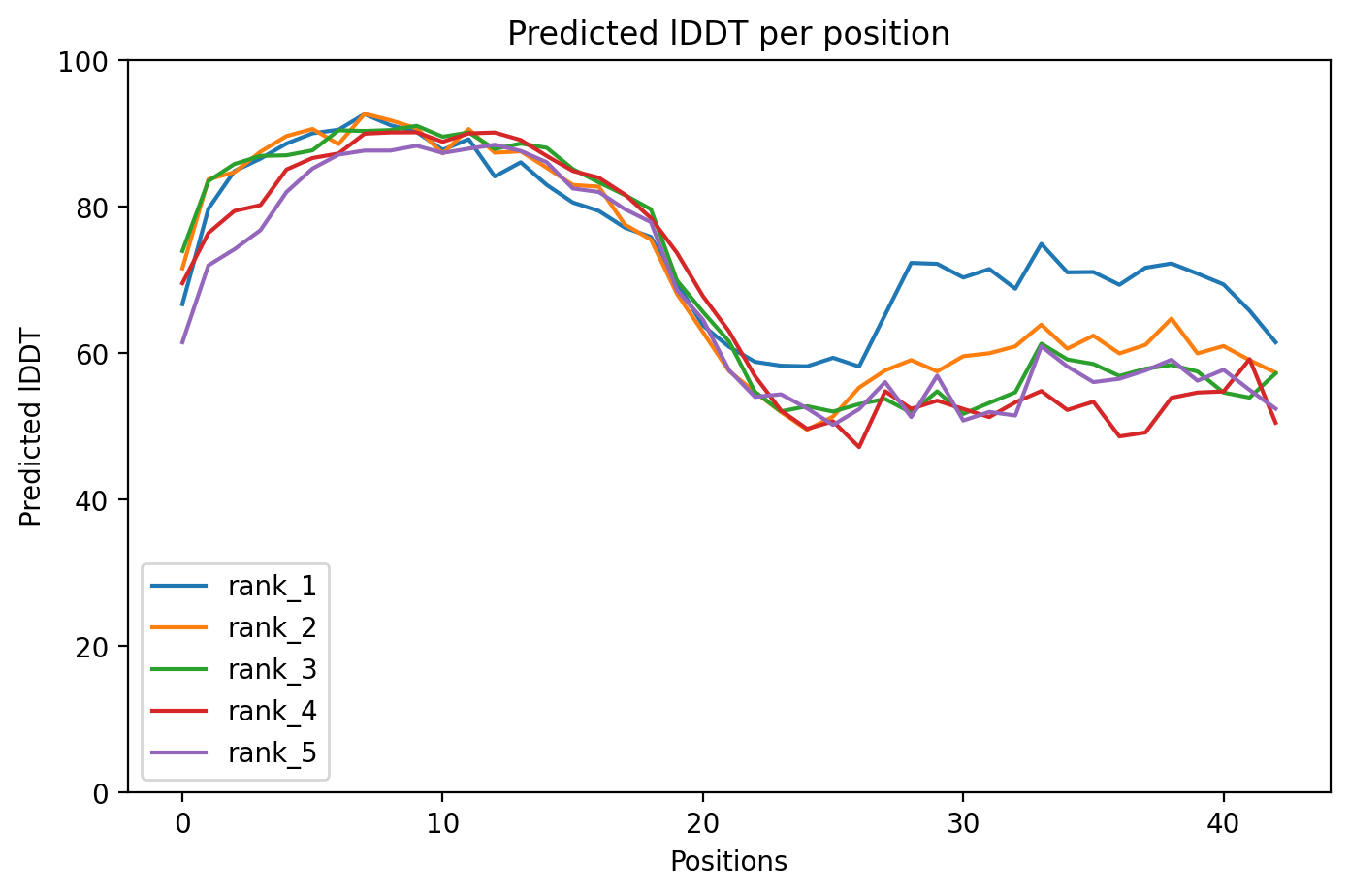

Supplement: lqaf178_Supplemental_Files [file lqaf178_supplemental_files.zip › BAGE1/predicted_LDDT.png]

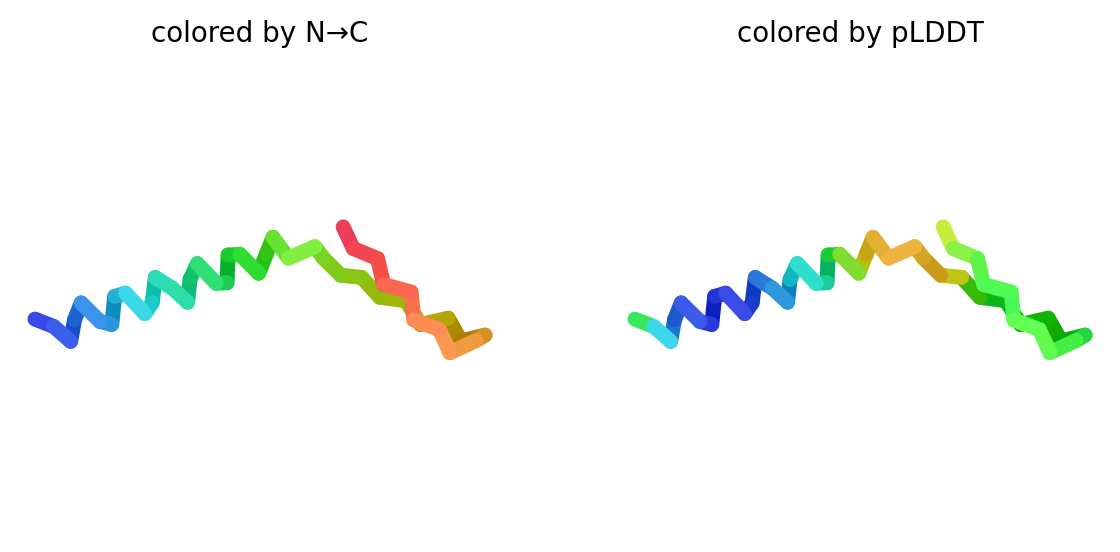

Supplement: lqaf178_Supplemental_Files [file lqaf178_supplemental_files.zip › BAGE1/rank_1_model_4_ptm_seed_0.png]

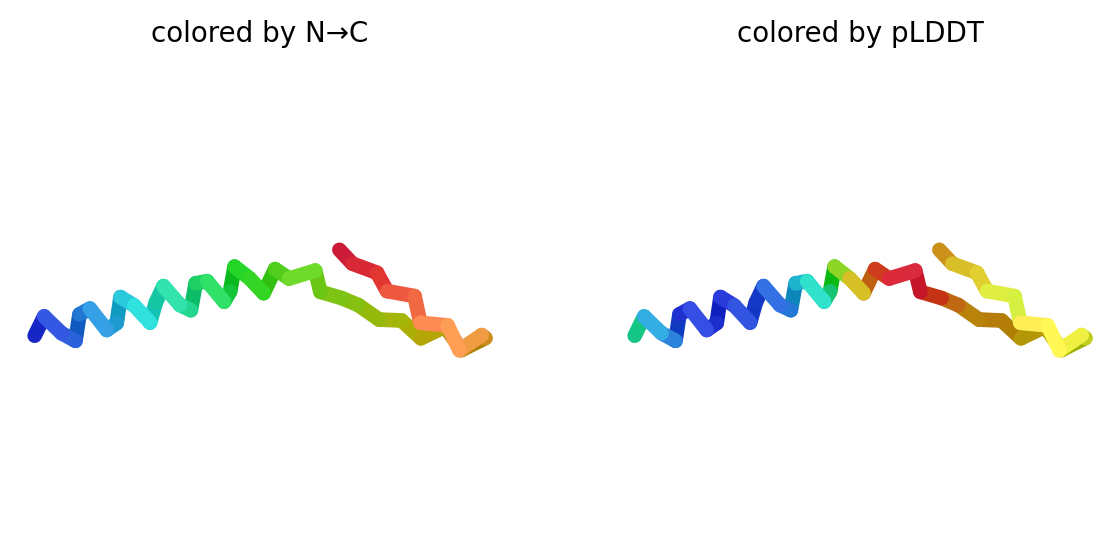

Supplement: lqaf178_Supplemental_Files [file lqaf178_supplemental_files.zip › BAGE1/rank_2_model_5_ptm_seed_0.png]

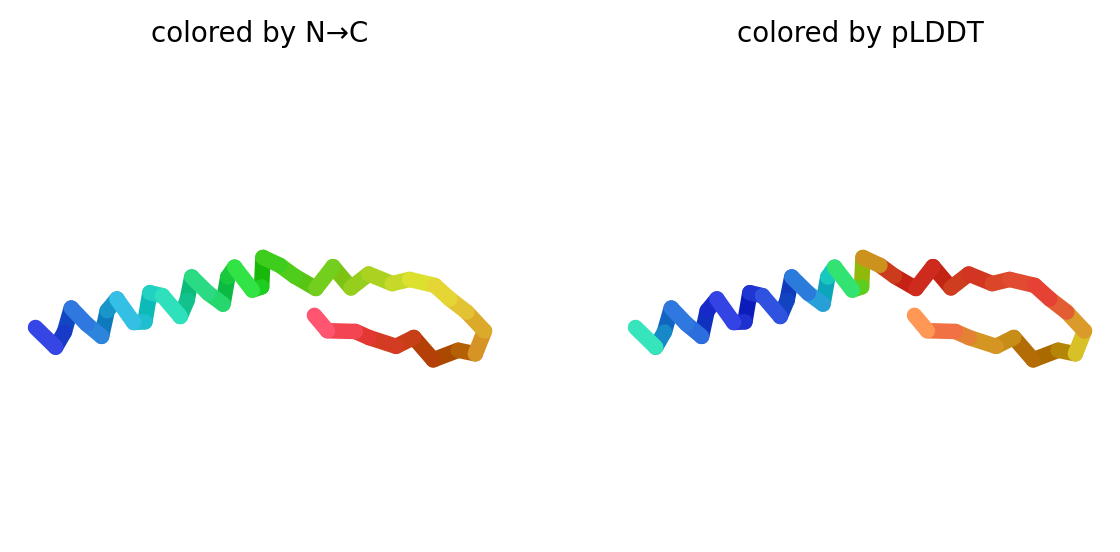

Supplement: lqaf178_Supplemental_Files [file lqaf178_supplemental_files.zip › BAGE1/rank_3_model_3_ptm_seed_0.png]

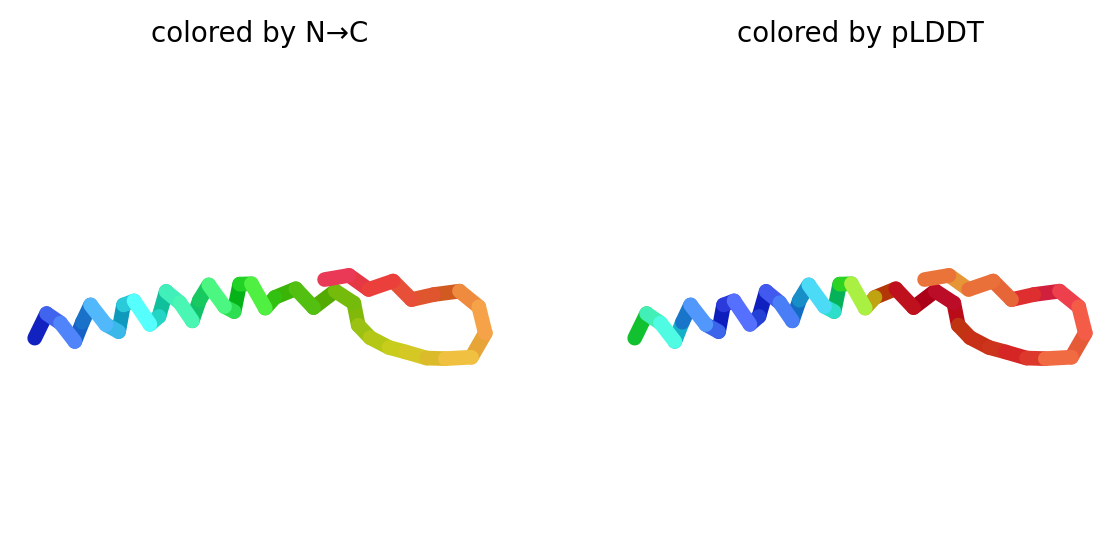

Supplement: lqaf178_Supplemental_Files [file lqaf178_supplemental_files.zip › BAGE1/rank_4_model_1_ptm_seed_0.png]

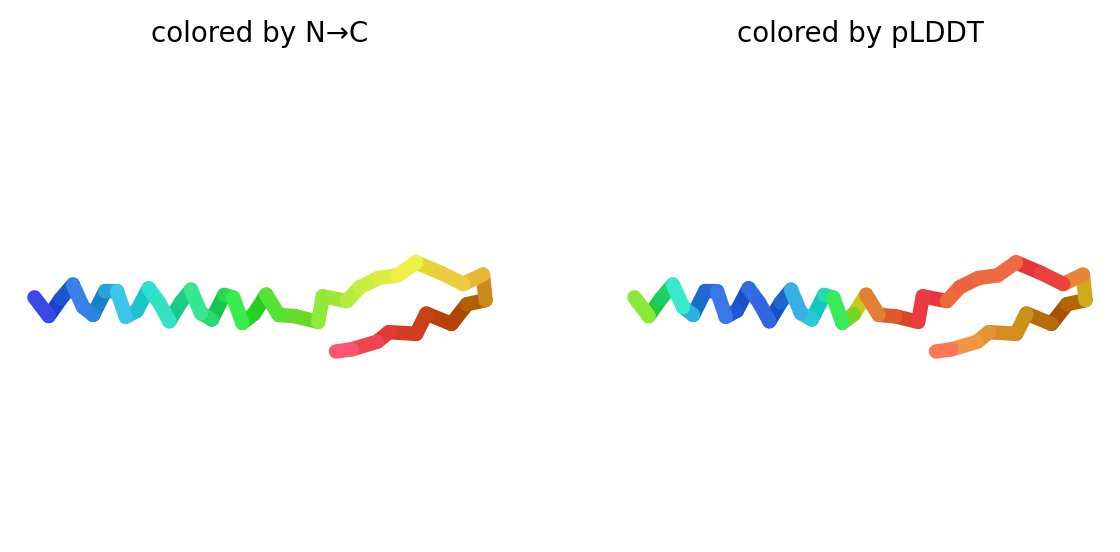

Supplement: lqaf178_Supplemental_Files [file lqaf178_supplemental_files.zip › BAGE1/rank_5_model_2_ptm_seed_0.png]

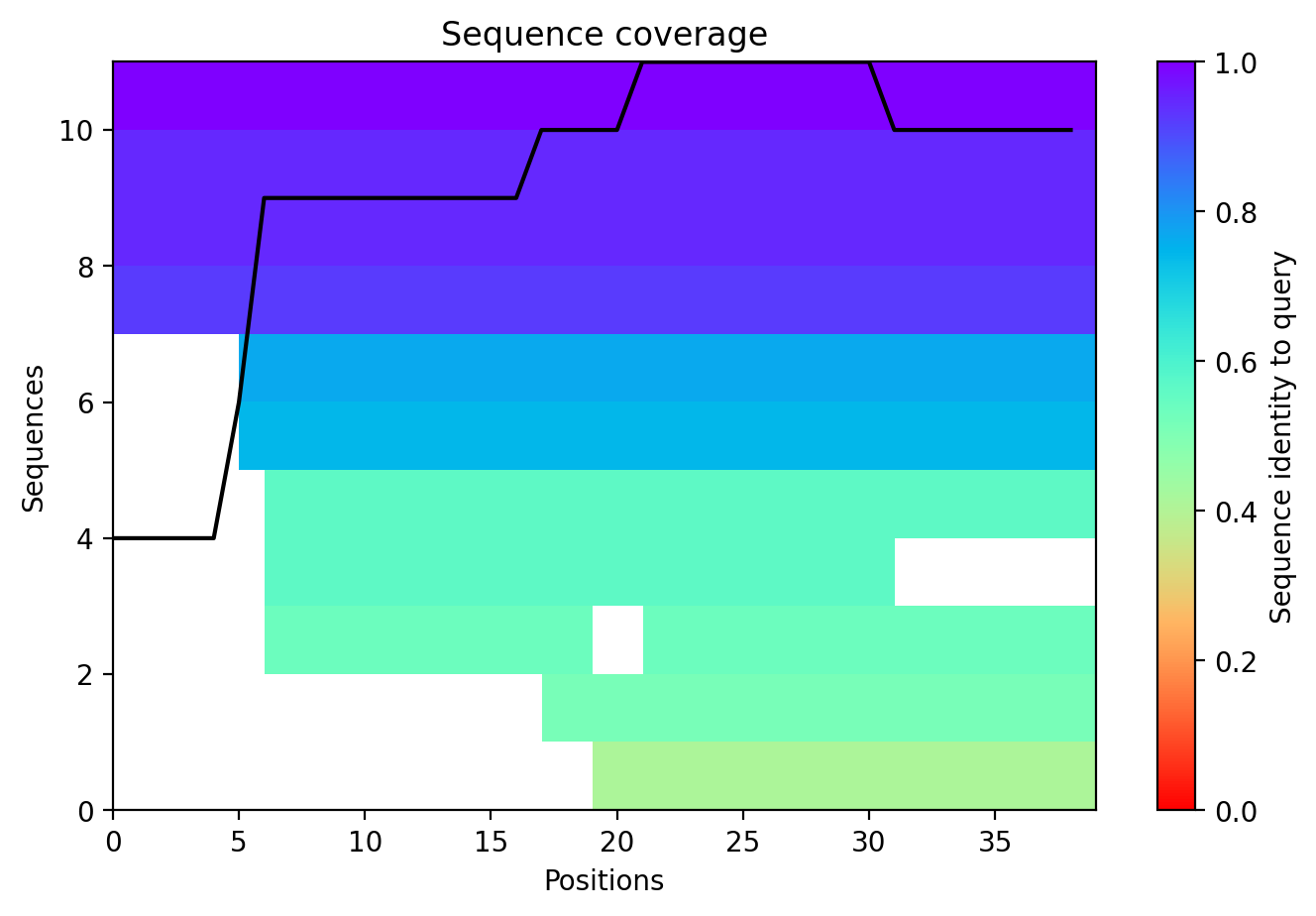

Supplement: lqaf178_Supplemental_Files [file lqaf178_supplemental_files.zip › BAGE4/msa_coverage.png]

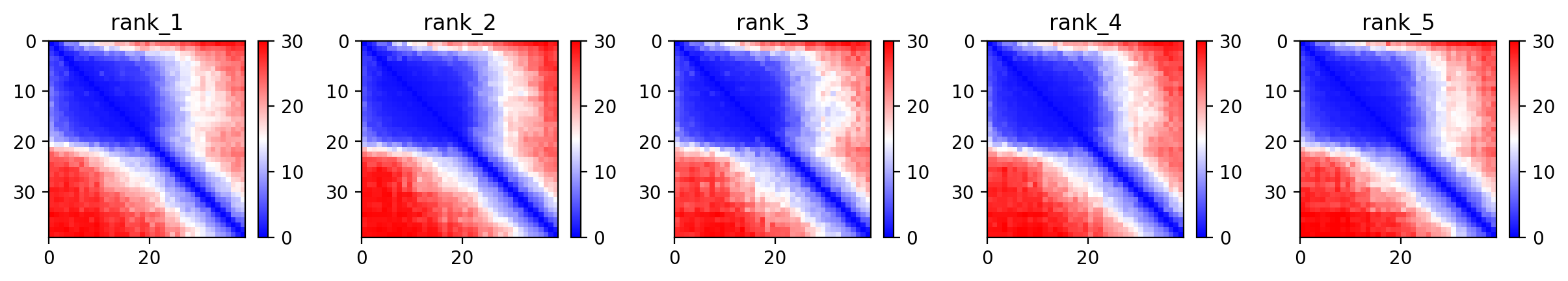

Supplement: lqaf178_Supplemental_Files [file lqaf178_supplemental_files.zip › BAGE4/predicted_alignment_error.png]

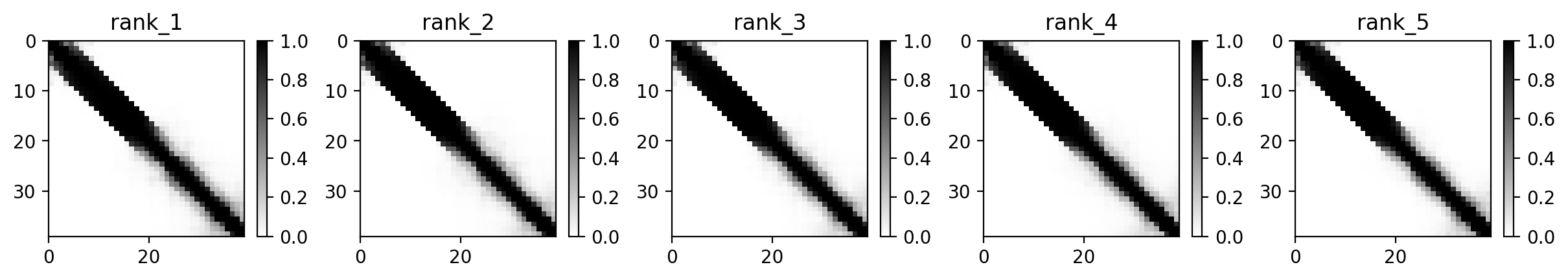

Supplement: lqaf178_Supplemental_Files [file lqaf178_supplemental_files.zip › BAGE4/predicted_contacts.png]

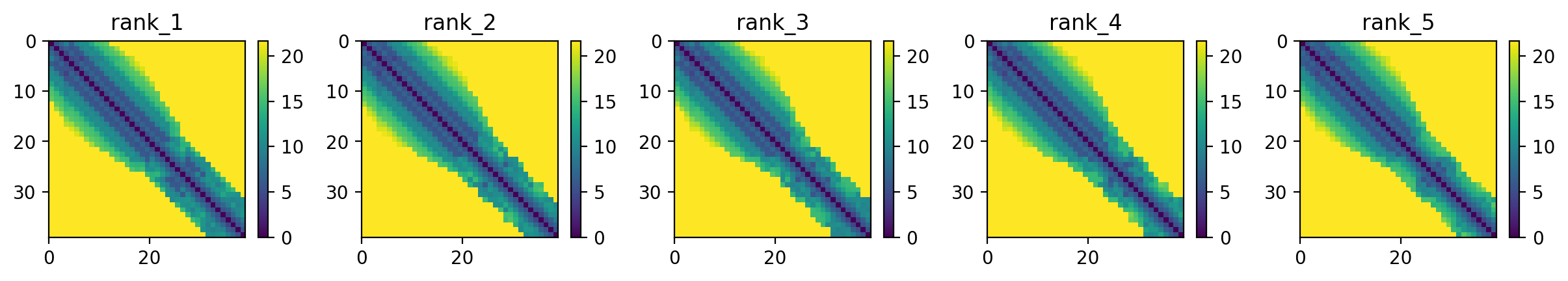

Supplement: lqaf178_Supplemental_Files [file lqaf178_supplemental_files.zip › BAGE4/predicted_distogram.png]

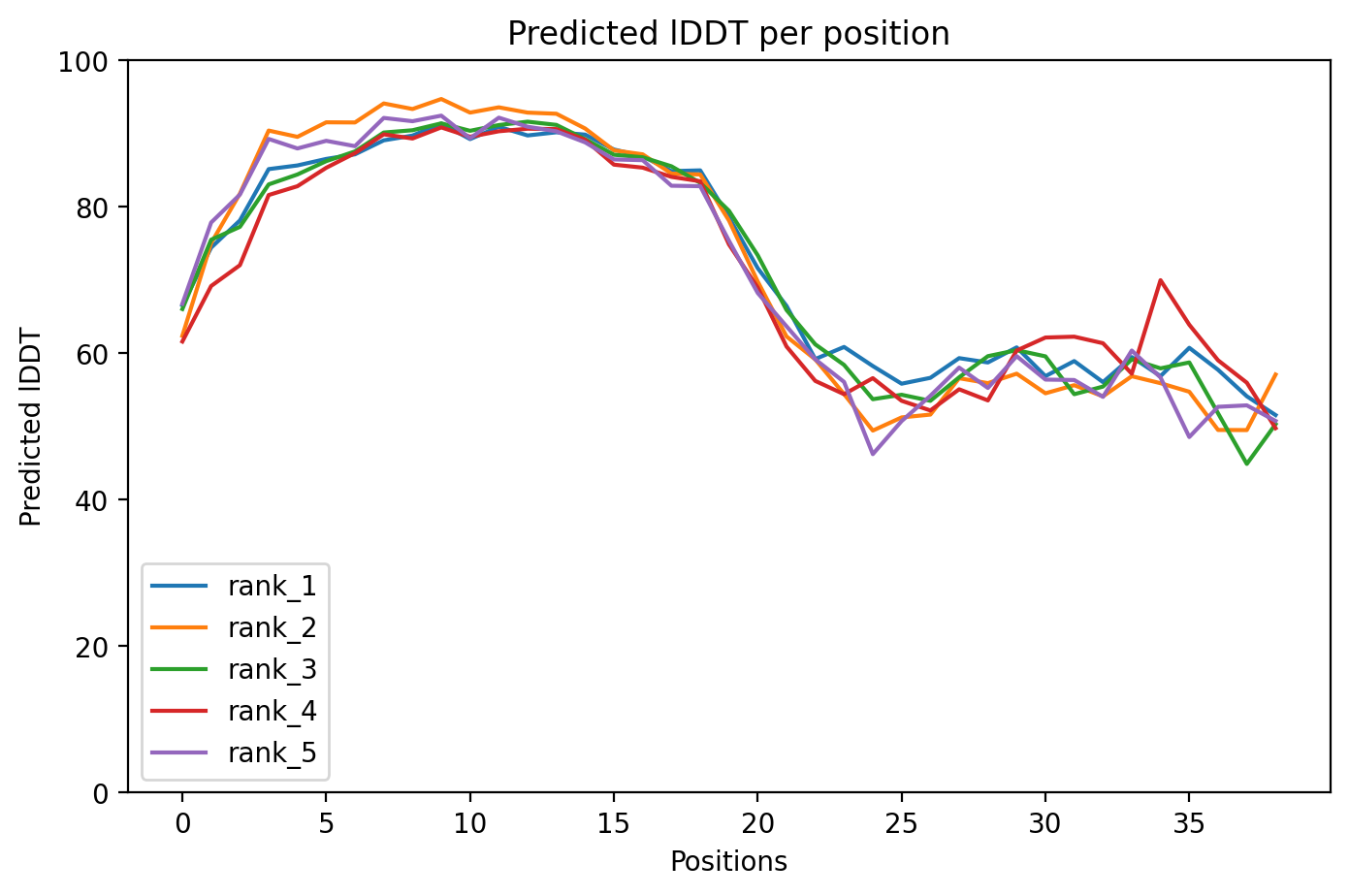

Supplement: lqaf178_Supplemental_Files [file lqaf178_supplemental_files.zip › BAGE4/predicted_LDDT.png]

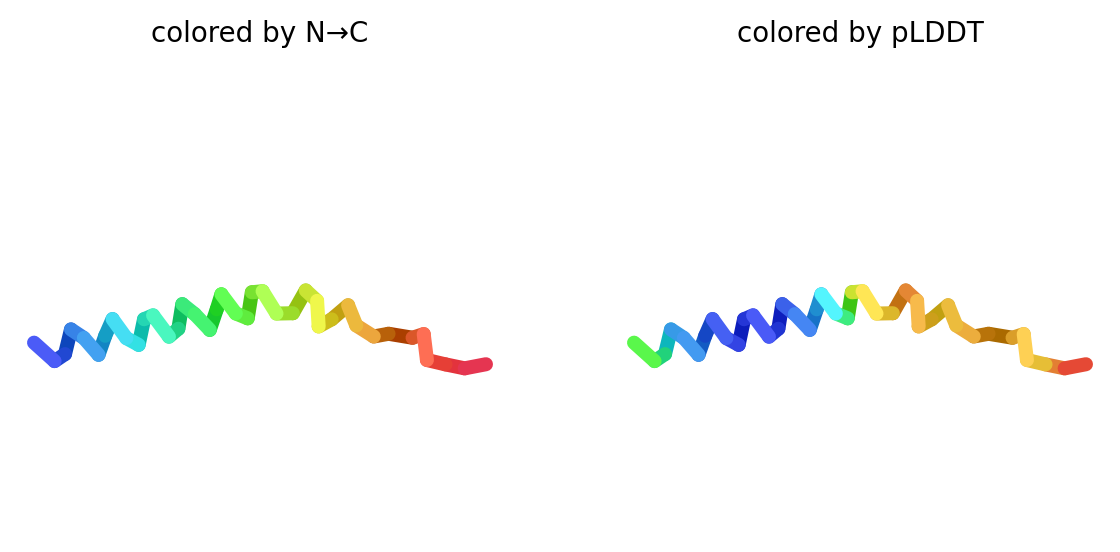

Supplement: lqaf178_Supplemental_Files [file lqaf178_supplemental_files.zip › BAGE4/rank_1_model_3_ptm_seed_0.png]

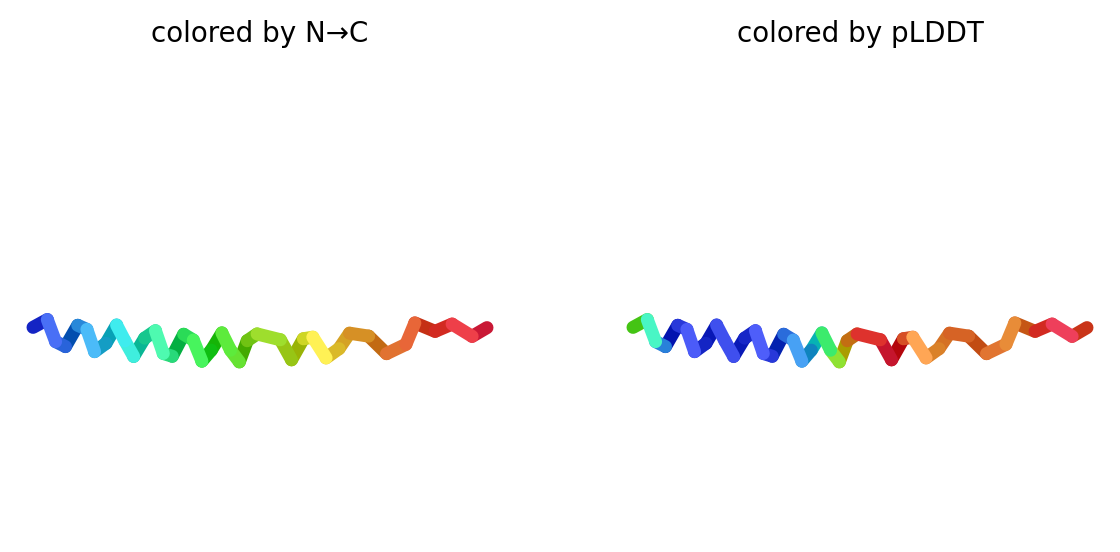

Supplement: lqaf178_Supplemental_Files [file lqaf178_supplemental_files.zip › BAGE4/rank_2_model_4_ptm_seed_0.png]

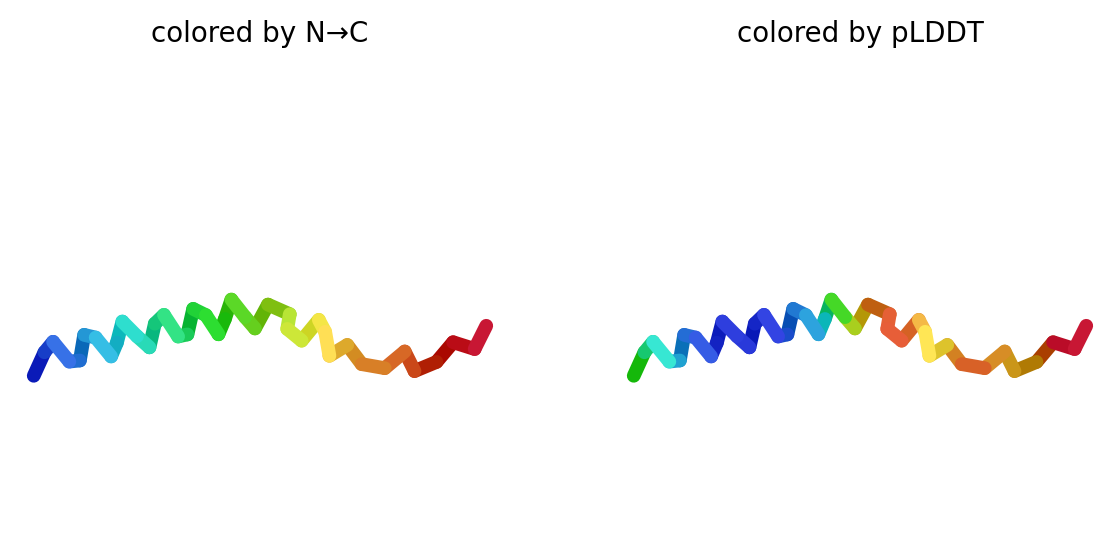

Supplement: lqaf178_Supplemental_Files [file lqaf178_supplemental_files.zip › BAGE4/rank_3_model_1_ptm_seed_0.png]

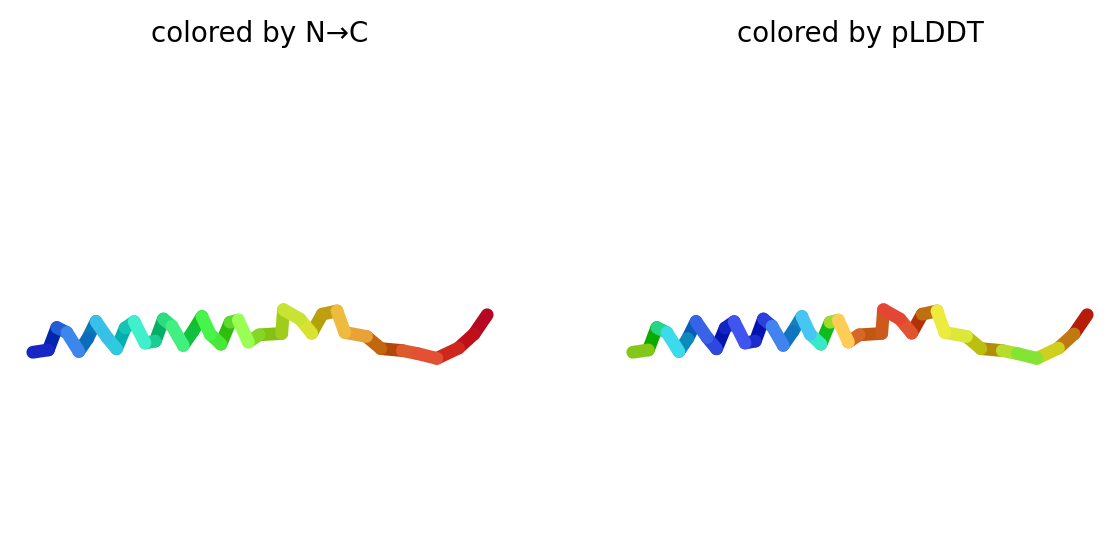

Supplement: lqaf178_Supplemental_Files [file lqaf178_supplemental_files.zip › BAGE4/rank_4_model_2_ptm_seed_0.png]

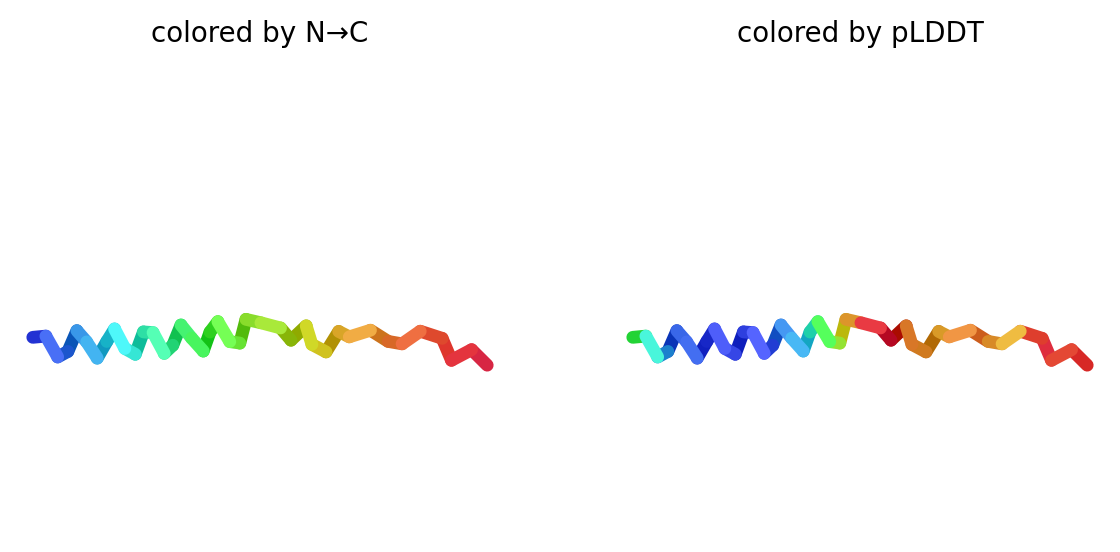

Supplement: lqaf178_Supplemental_Files [file lqaf178_supplemental_files.zip › BAGE4/rank_5_model_5_ptm_seed_0.png]

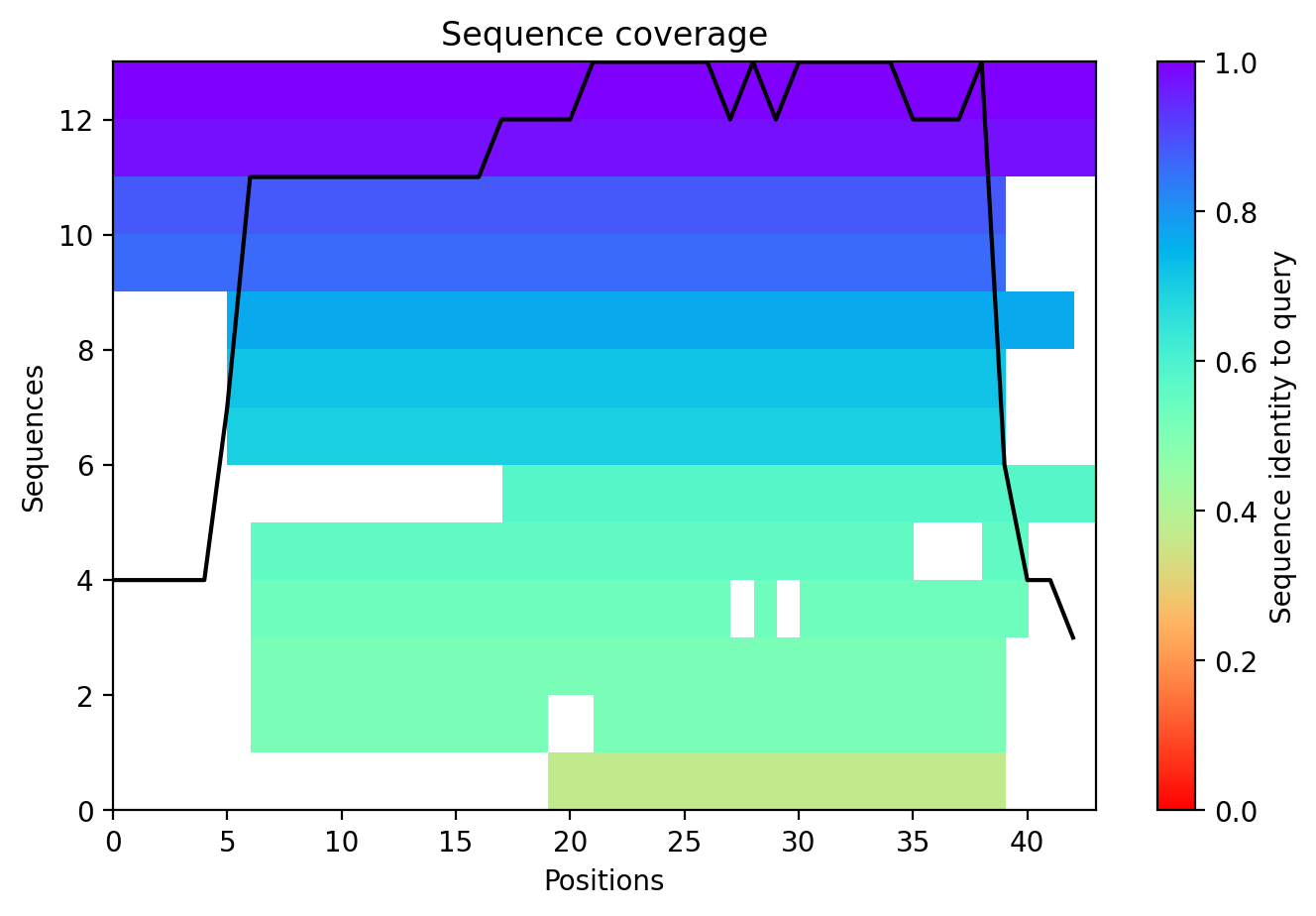

Supplement: lqaf178_Supplemental_Files [file lqaf178_supplemental_files.zip › BAGE5/msa_coverage.png]

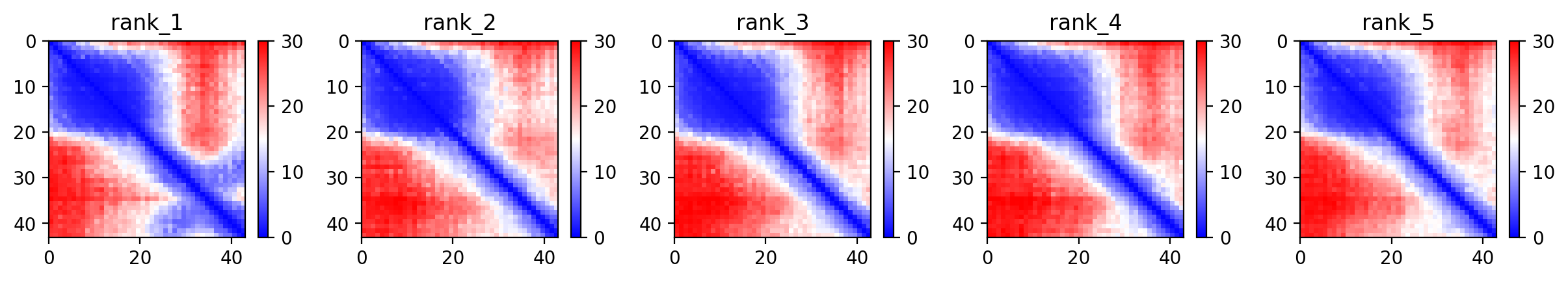

Supplement: lqaf178_Supplemental_Files [file lqaf178_supplemental_files.zip › BAGE5/predicted_alignment_error.png]

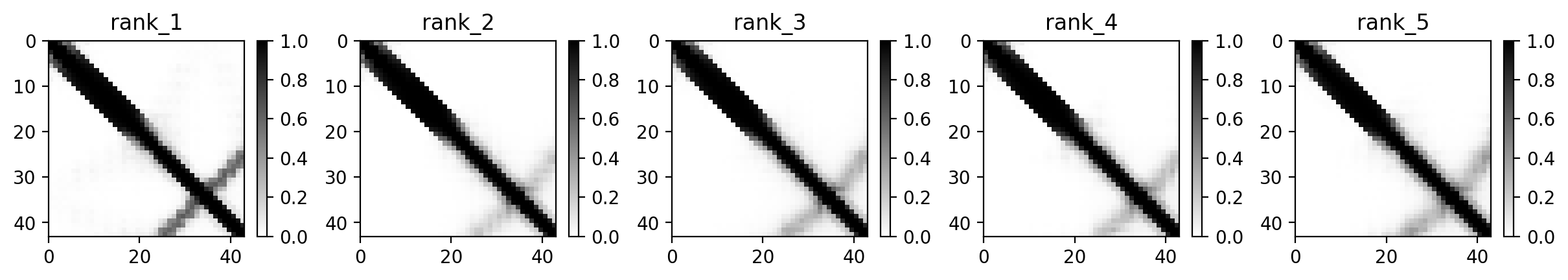

Supplement: lqaf178_Supplemental_Files [file lqaf178_supplemental_files.zip › BAGE5/predicted_contacts.png]

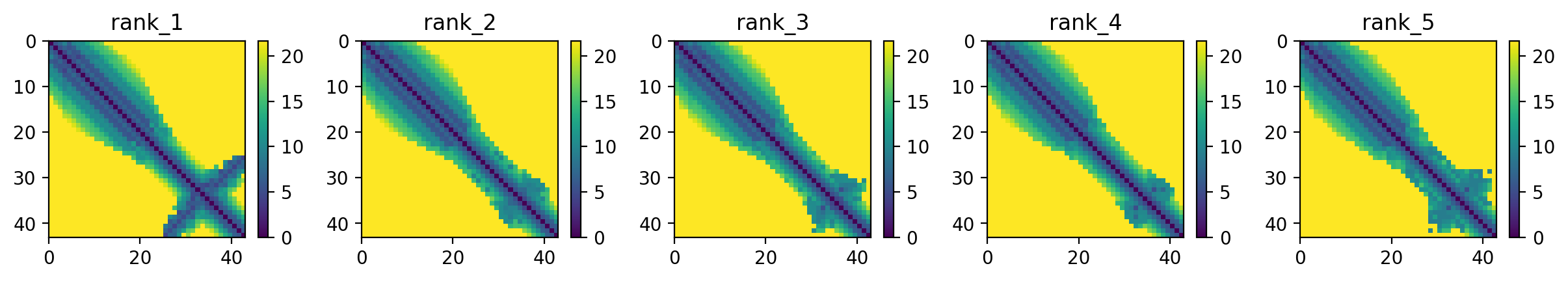

Supplement: lqaf178_Supplemental_Files [file lqaf178_supplemental_files.zip › BAGE5/predicted_distogram.png]

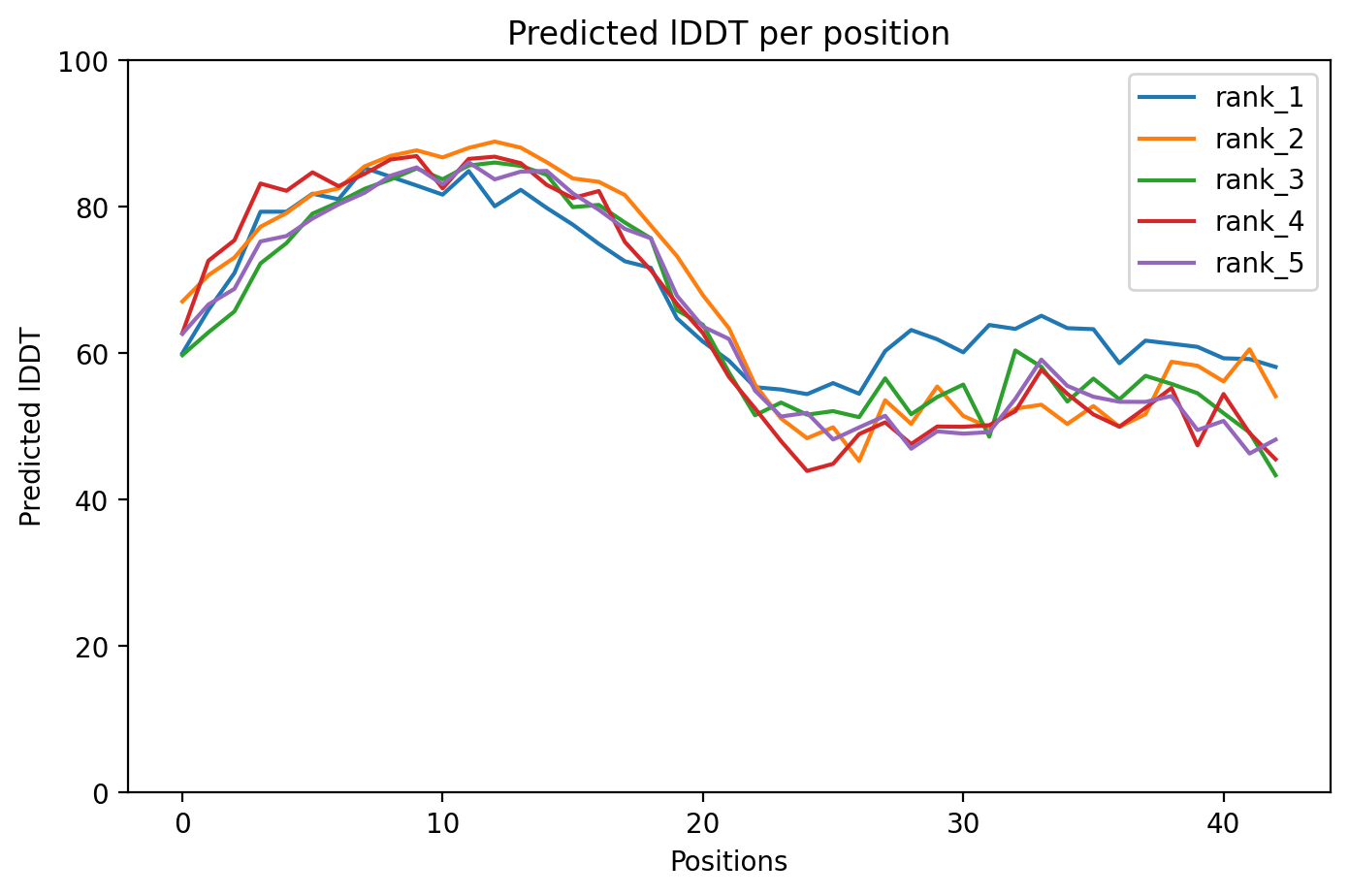

Supplement: lqaf178_Supplemental_Files [file lqaf178_supplemental_files.zip › BAGE5/predicted_LDDT.png]

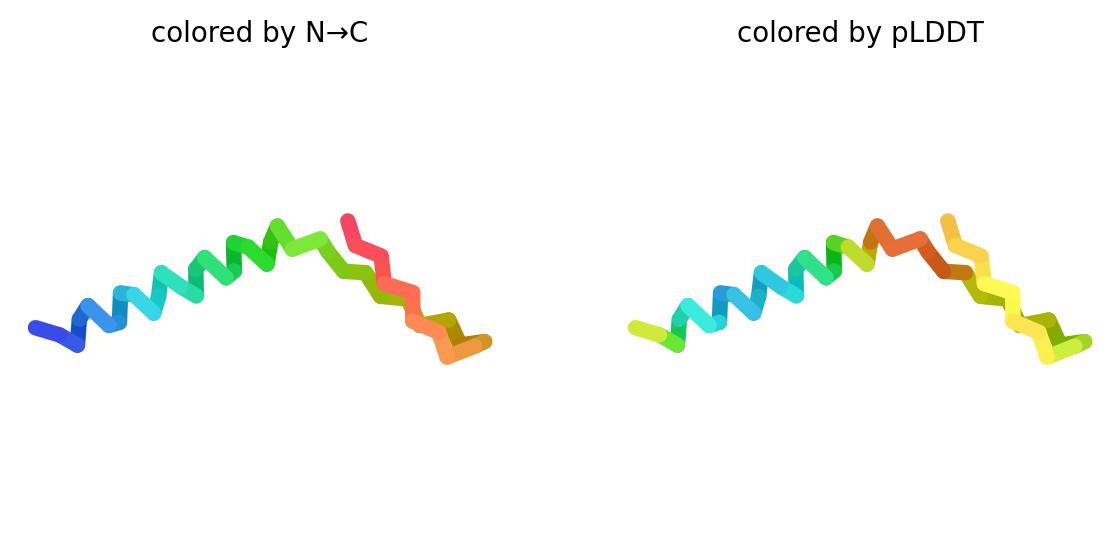

Supplement: lqaf178_Supplemental_Files [file lqaf178_supplemental_files.zip › BAGE5/rank_1_model_4_ptm_seed_0.png]

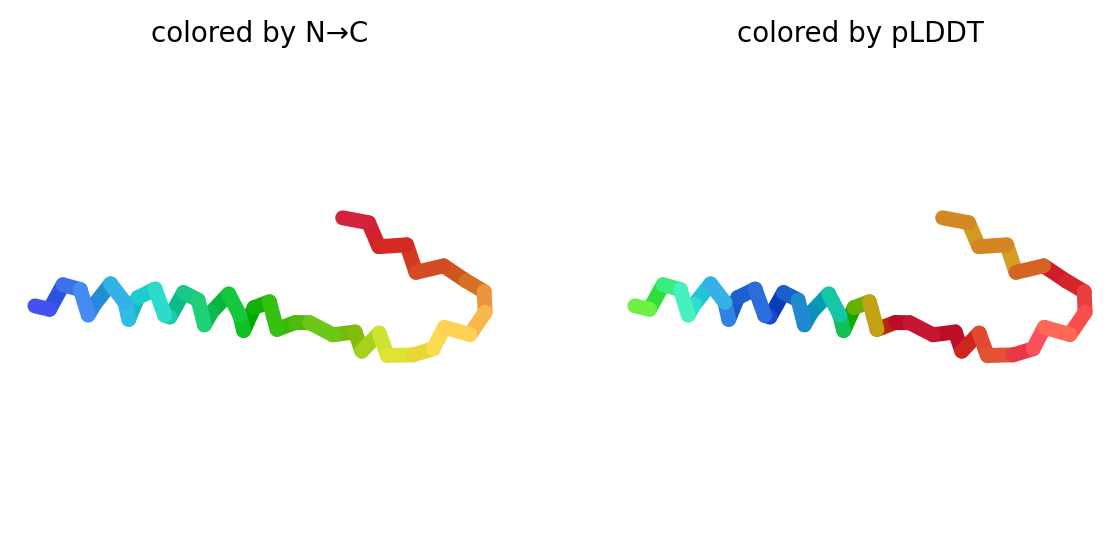

Supplement: lqaf178_Supplemental_Files [file lqaf178_supplemental_files.zip › BAGE5/rank_2_model_1_ptm_seed_0.png]

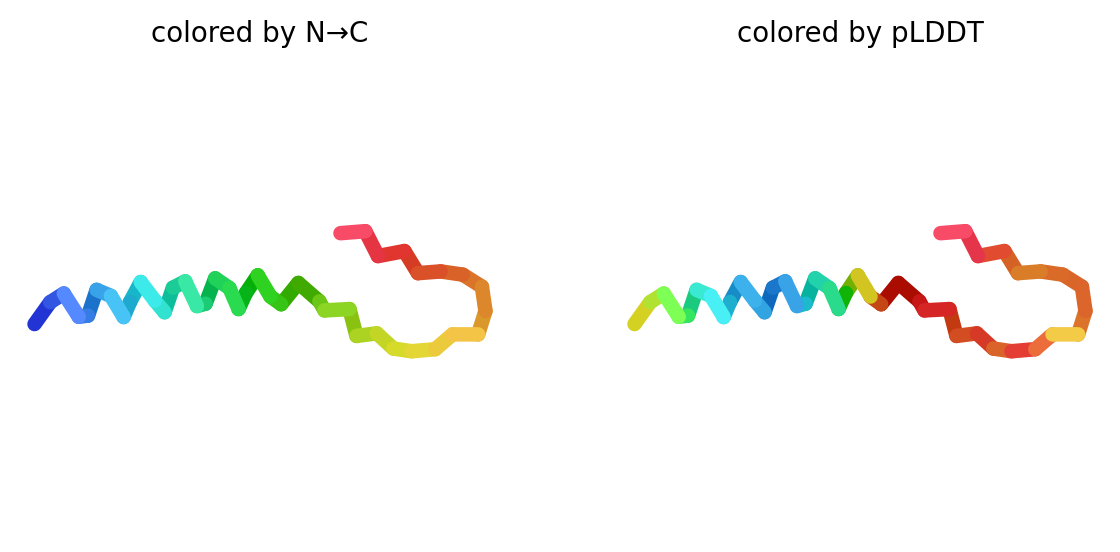

Supplement: lqaf178_Supplemental_Files [file lqaf178_supplemental_files.zip › BAGE5/rank_3_model_2_ptm_seed_0.png]

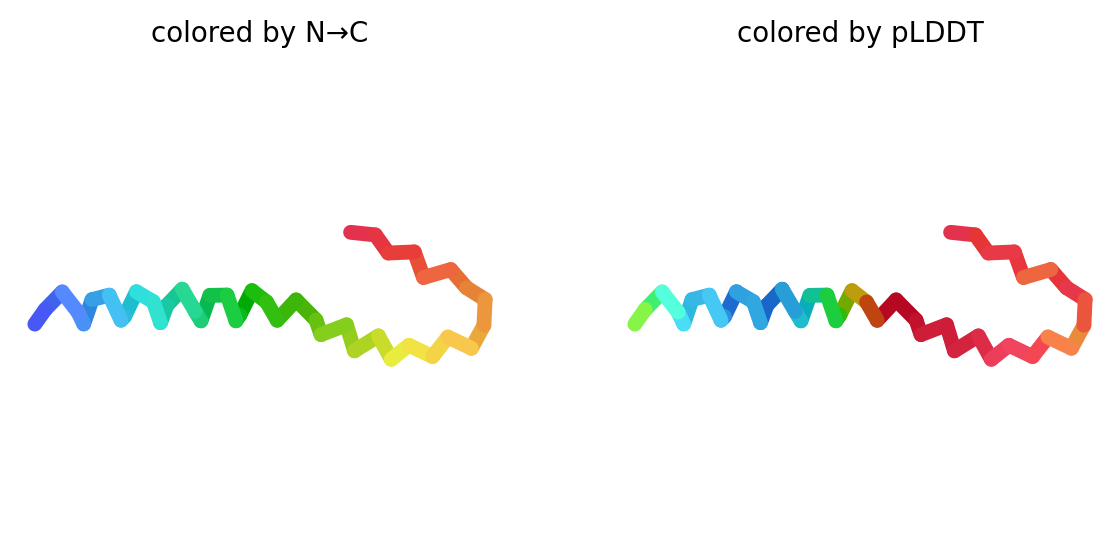

Supplement: lqaf178_Supplemental_Files [file lqaf178_supplemental_files.zip › BAGE5/rank_4_model_5_ptm_seed_0.png]

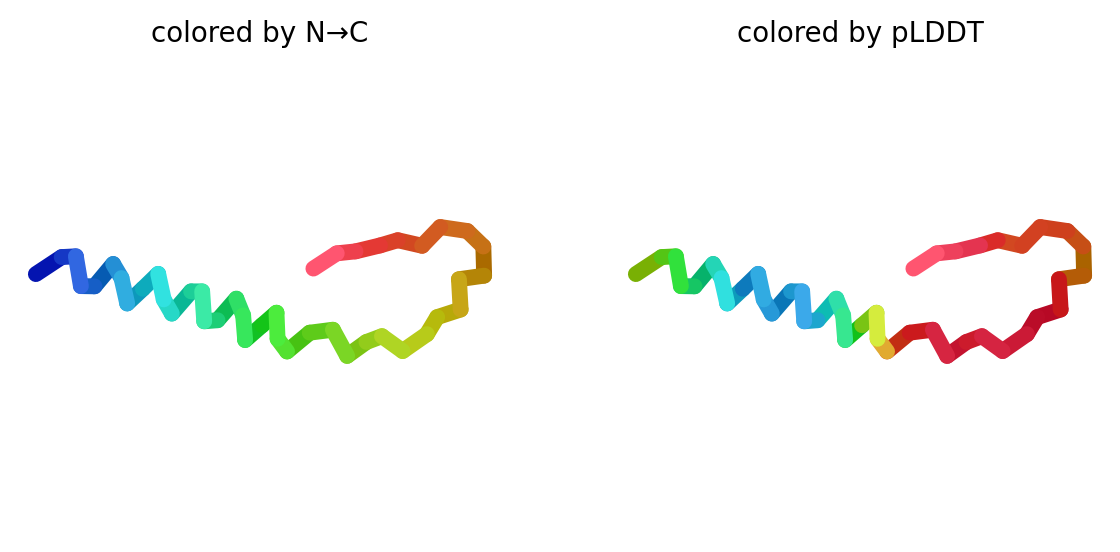

Supplement: lqaf178_Supplemental_Files [file lqaf178_supplemental_files.zip › BAGE5/rank_5_model_3_ptm_seed_0.png]

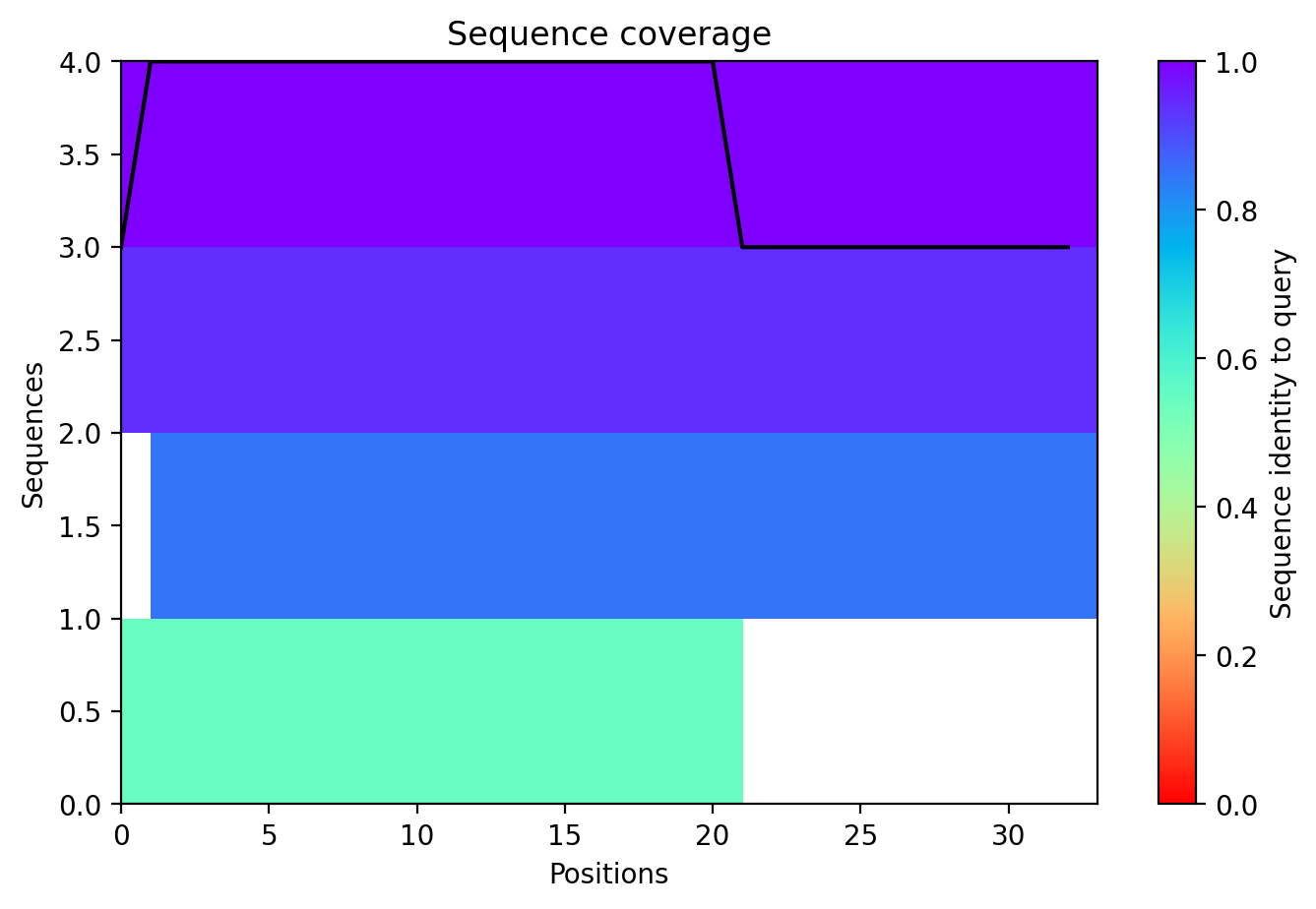

Supplement: lqaf178_Supplemental_Files [file lqaf178_supplemental_files.zip › CT18/msa_coverage.png]

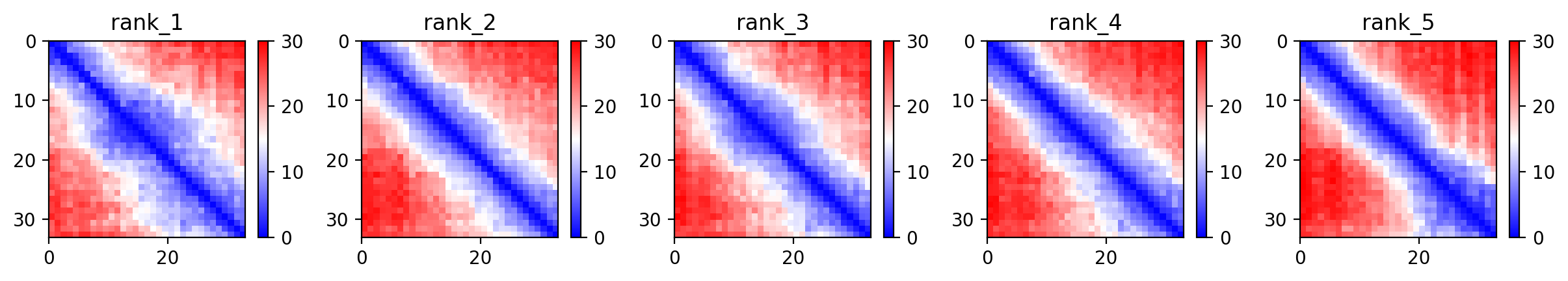

Supplement: lqaf178_Supplemental_Files [file lqaf178_supplemental_files.zip › CT18/predicted_alignment_error.png]

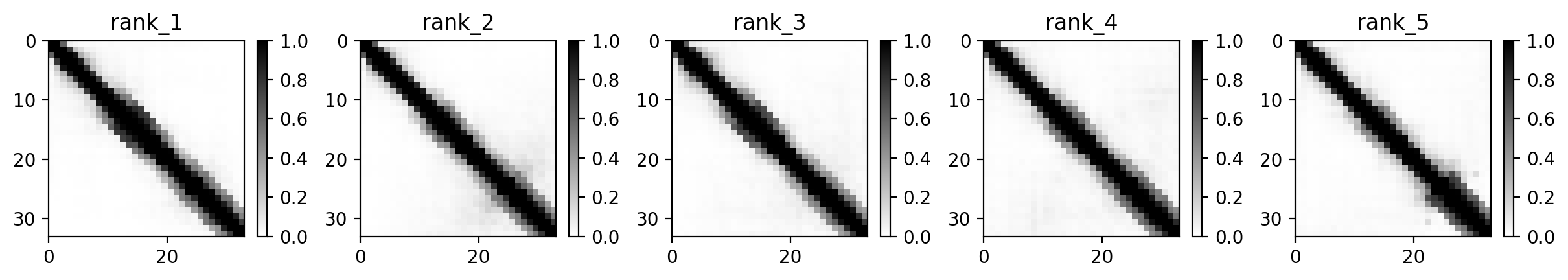

Supplement: lqaf178_Supplemental_Files [file lqaf178_supplemental_files.zip › CT18/predicted_contacts.png]

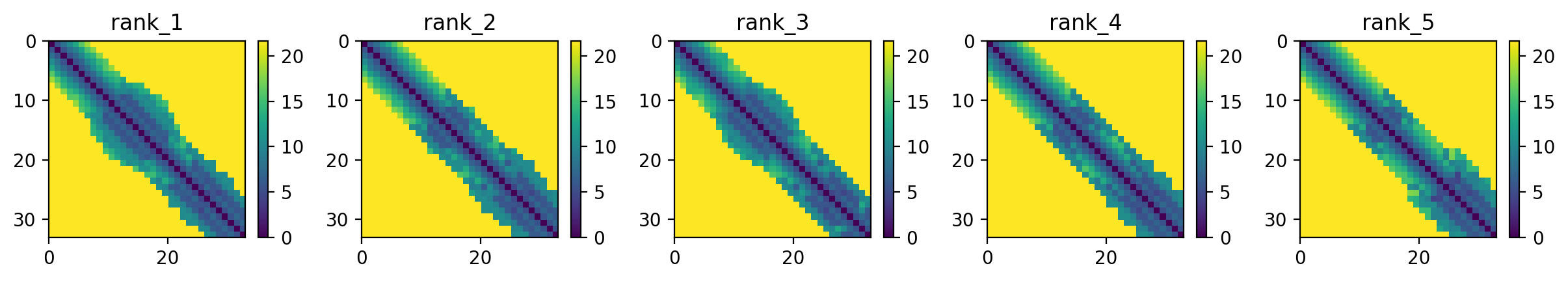

Supplement: lqaf178_Supplemental_Files [file lqaf178_supplemental_files.zip › CT18/predicted_distogram.png]

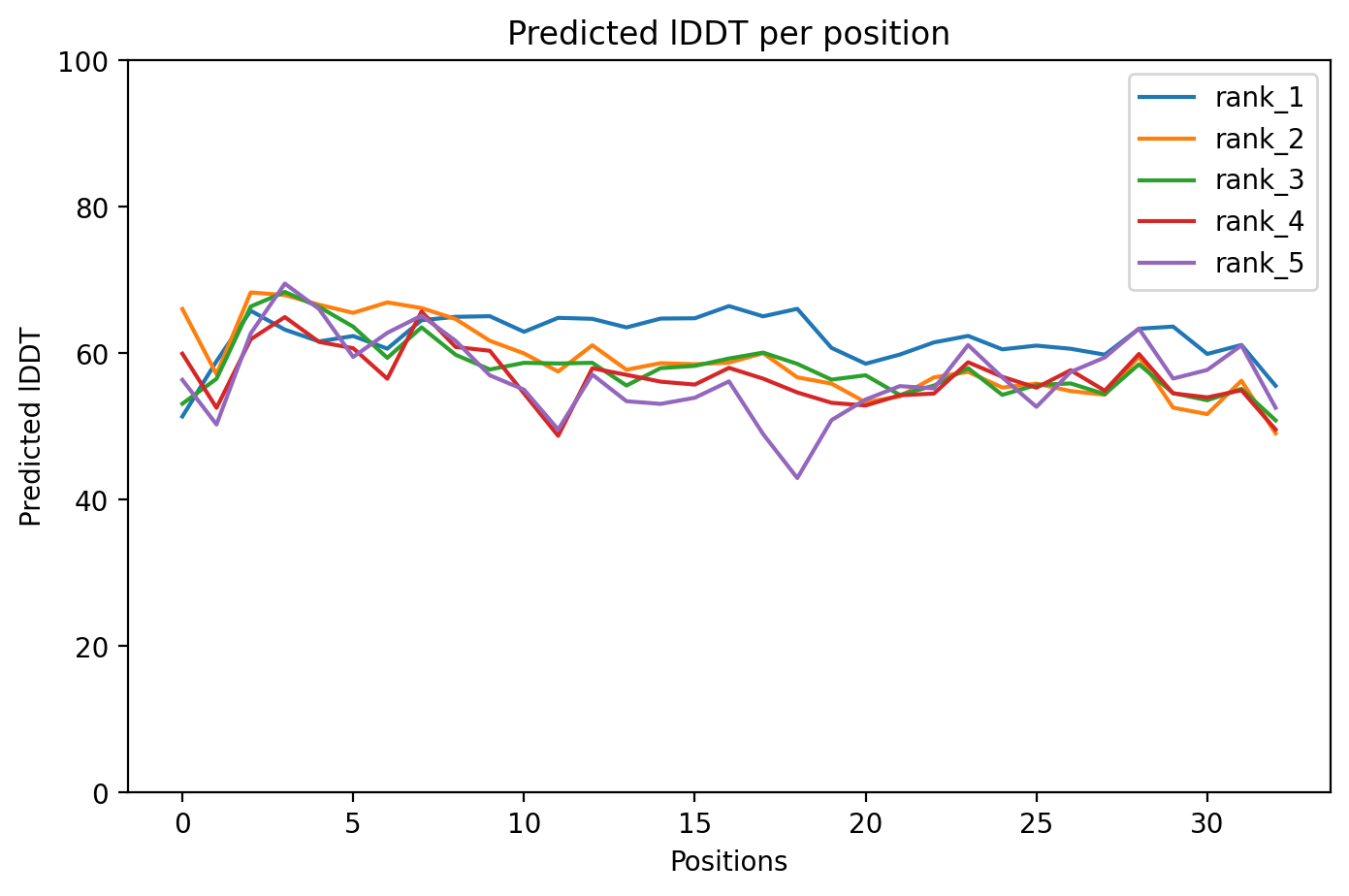

Supplement: lqaf178_Supplemental_Files [file lqaf178_supplemental_files.zip › CT18/predicted_LDDT.png]

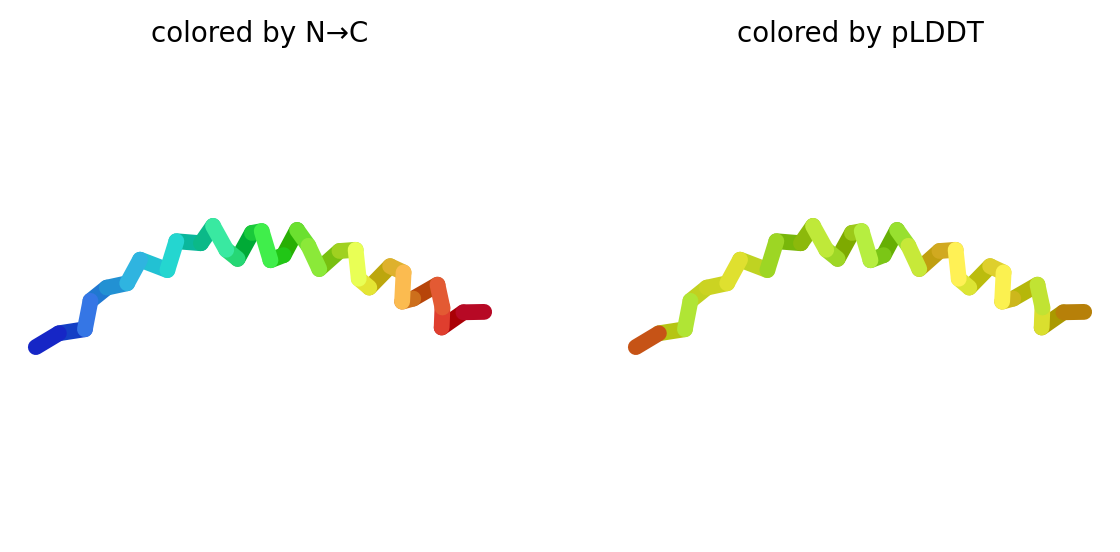

Supplement: lqaf178_Supplemental_Files [file lqaf178_supplemental_files.zip › CT18/rank_1_model_1_ptm_seed_0.png]

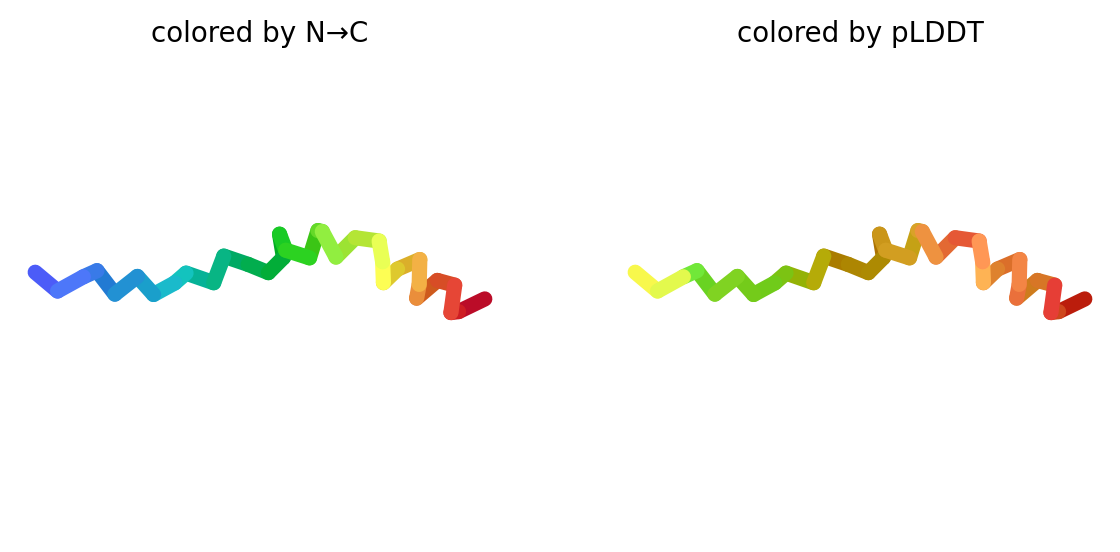

Supplement: lqaf178_Supplemental_Files [file lqaf178_supplemental_files.zip › CT18/rank_2_model_3_ptm_seed_0.png]

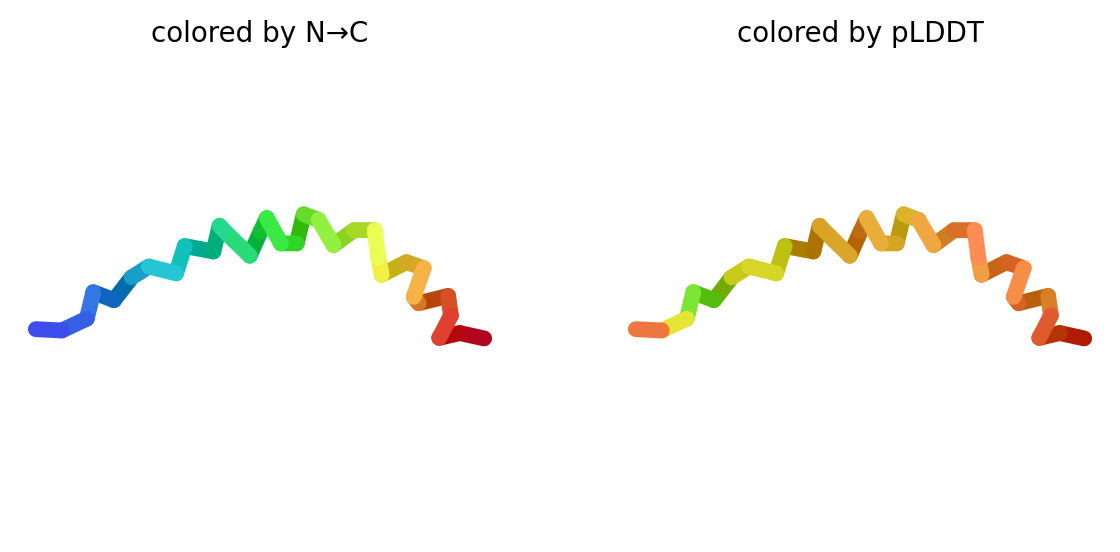

Supplement: lqaf178_Supplemental_Files [file lqaf178_supplemental_files.zip › CT18/rank_3_model_2_ptm_seed_0.png]

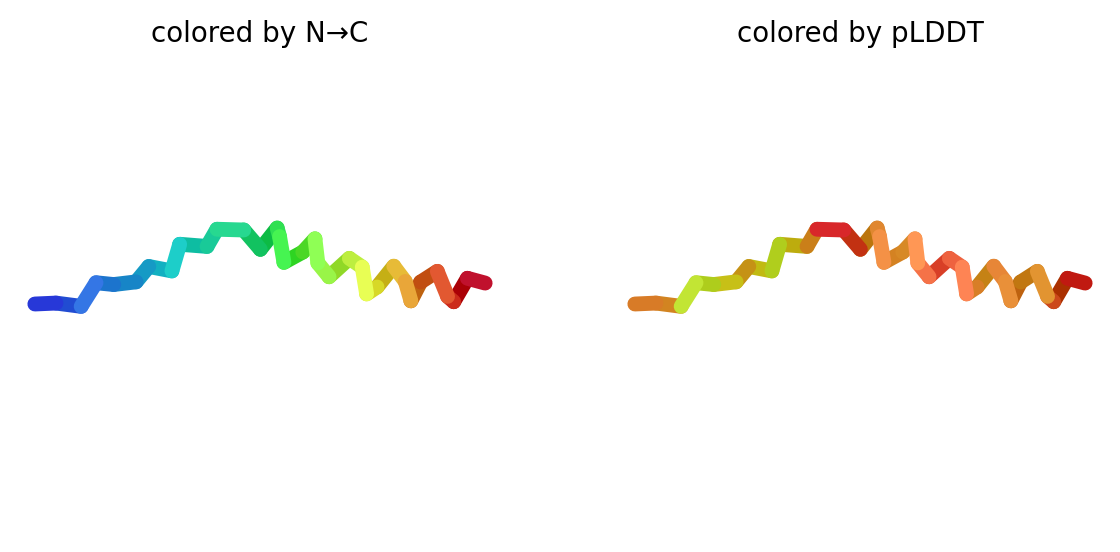

Supplement: lqaf178_Supplemental_Files [file lqaf178_supplemental_files.zip › CT18/rank_4_model_4_ptm_seed_0.png]

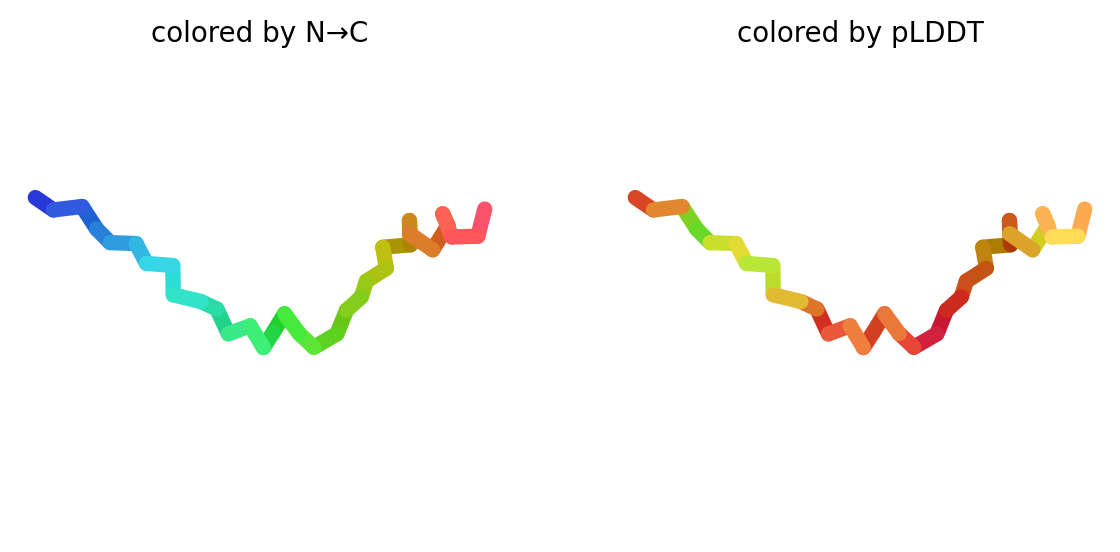

Supplement: lqaf178_Supplemental_Files [file lqaf178_supplemental_files.zip › CT18/rank_5_model_5_ptm_seed_0.png]

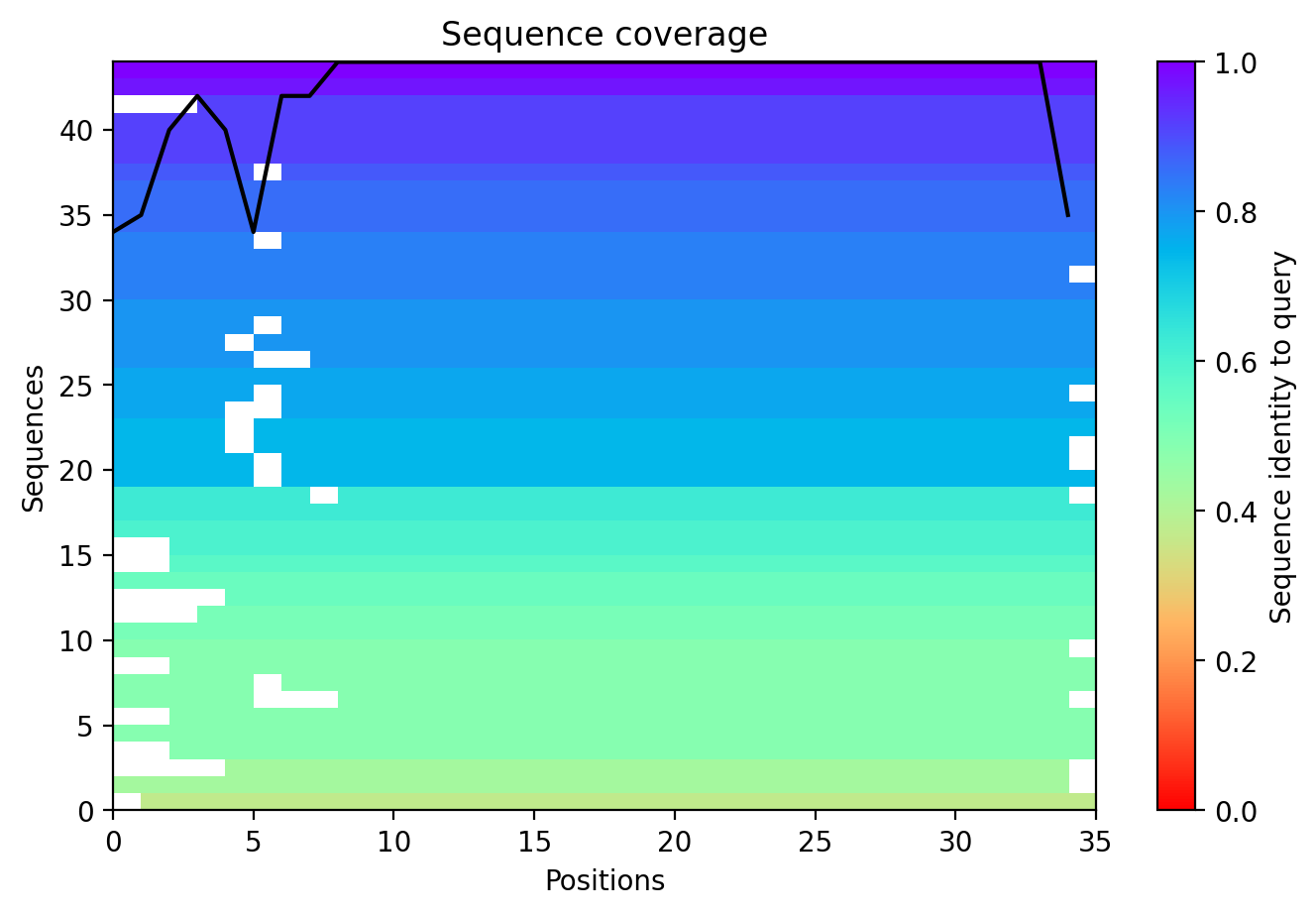

Supplement: lqaf178_Supplemental_Files [file lqaf178_supplemental_files.zip › DWORF/msa_coverage.png]

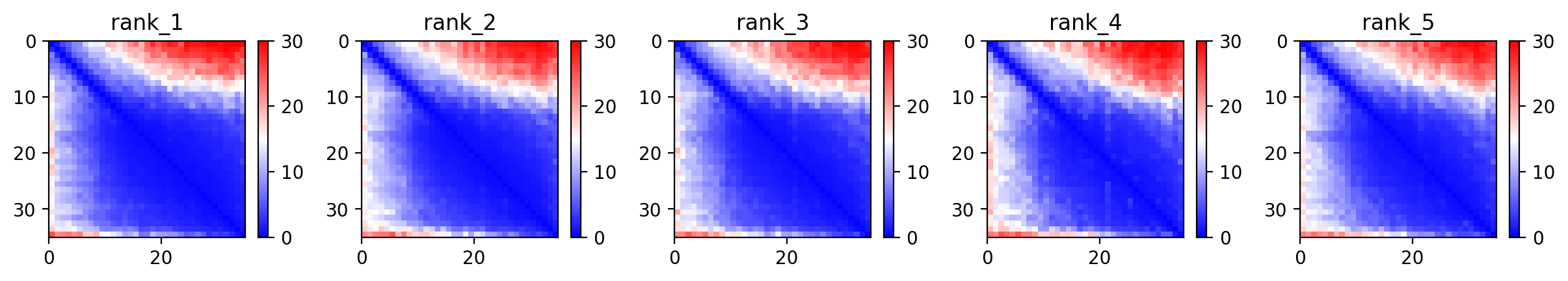

Supplement: lqaf178_Supplemental_Files [file lqaf178_supplemental_files.zip › DWORF/predicted_alignment_error.png]

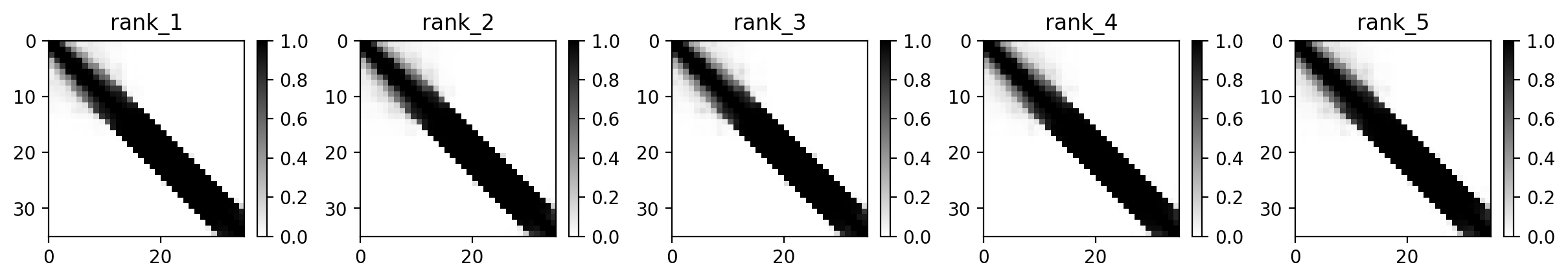

Supplement: lqaf178_Supplemental_Files [file lqaf178_supplemental_files.zip › DWORF/predicted_contacts.png]

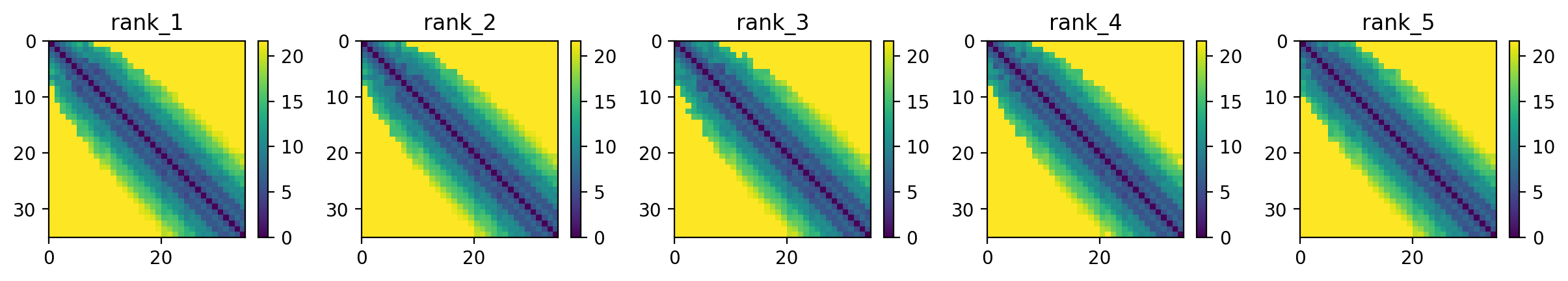

Supplement: lqaf178_Supplemental_Files [file lqaf178_supplemental_files.zip › DWORF/predicted_distogram.png]

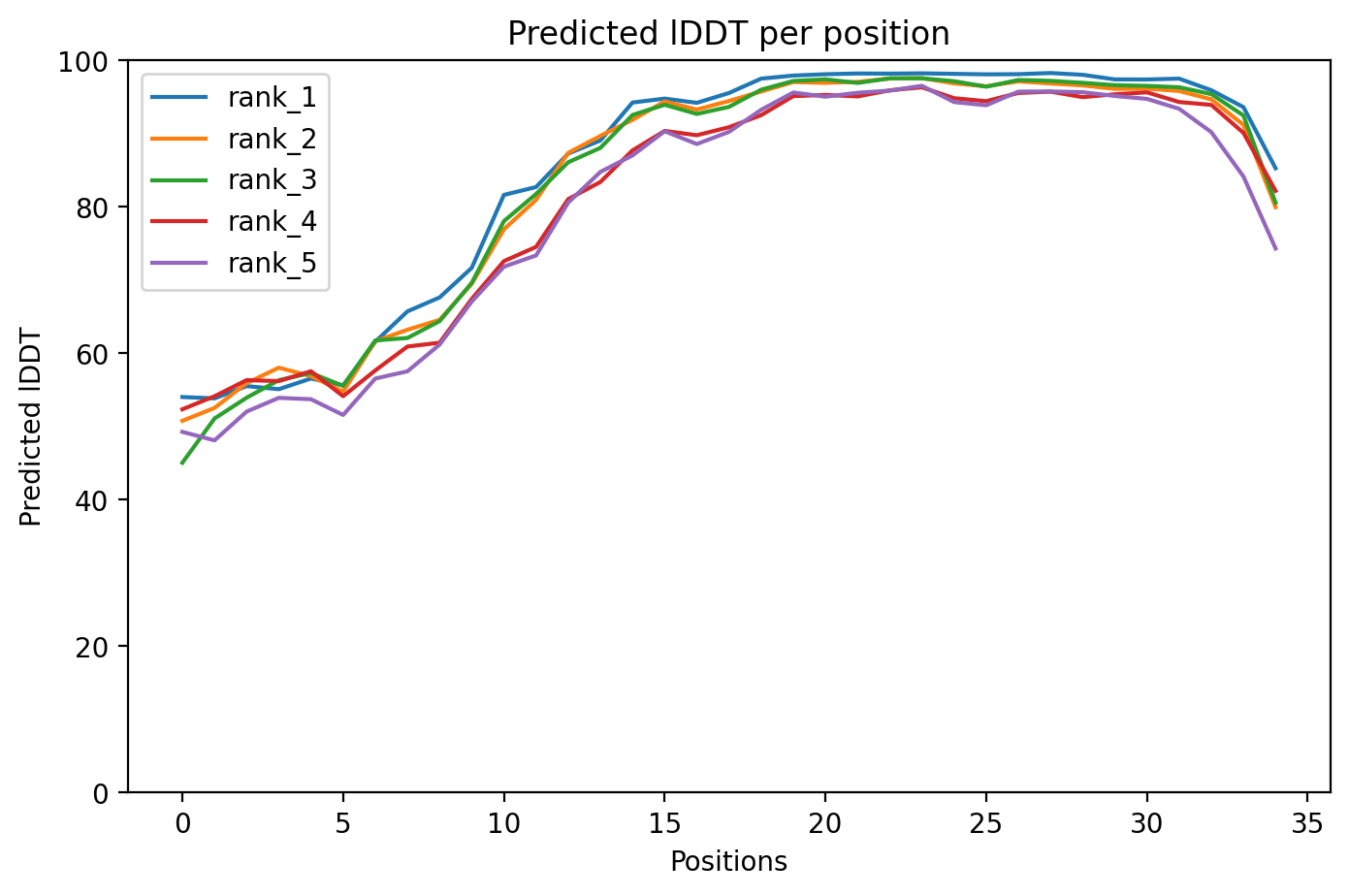

Supplement: lqaf178_Supplemental_Files [file lqaf178_supplemental_files.zip › DWORF/predicted_LDDT.png]

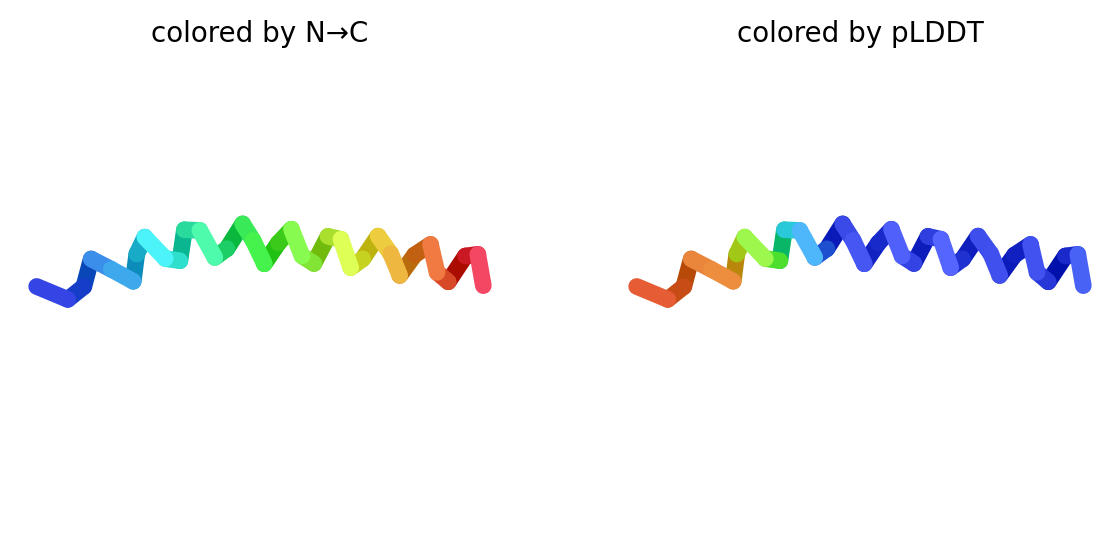

Supplement: lqaf178_Supplemental_Files [file lqaf178_supplemental_files.zip › DWORF/rank_1_model_5_ptm_seed_0.png]

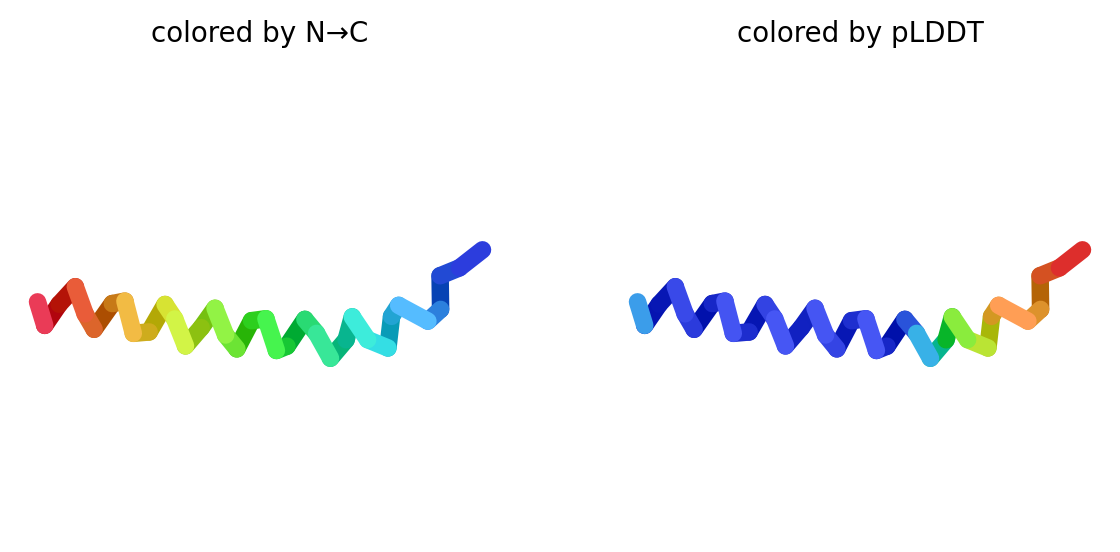

Supplement: lqaf178_Supplemental_Files [file lqaf178_supplemental_files.zip › DWORF/rank_2_model_3_ptm_seed_0.png]

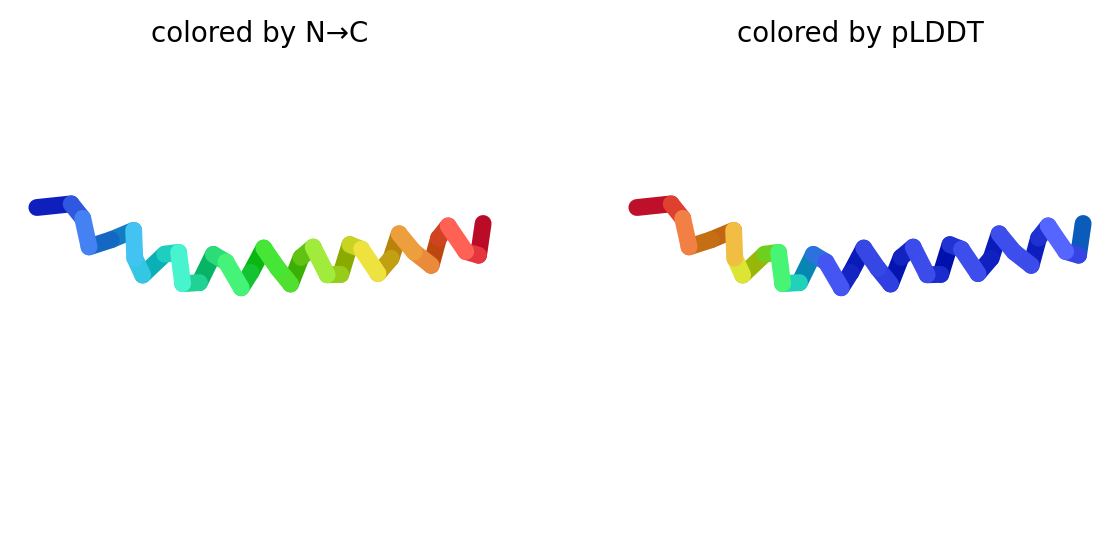

Supplement: lqaf178_Supplemental_Files [file lqaf178_supplemental_files.zip › DWORF/rank_3_model_4_ptm_seed_0.png]

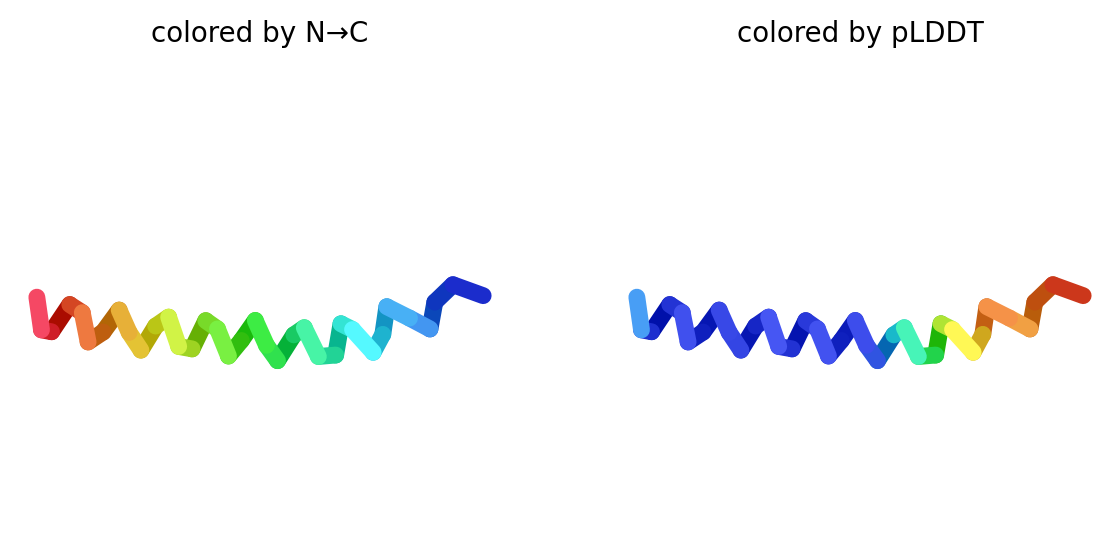

Supplement: lqaf178_Supplemental_Files [file lqaf178_supplemental_files.zip › DWORF/rank_4_model_1_ptm_seed_0.png]

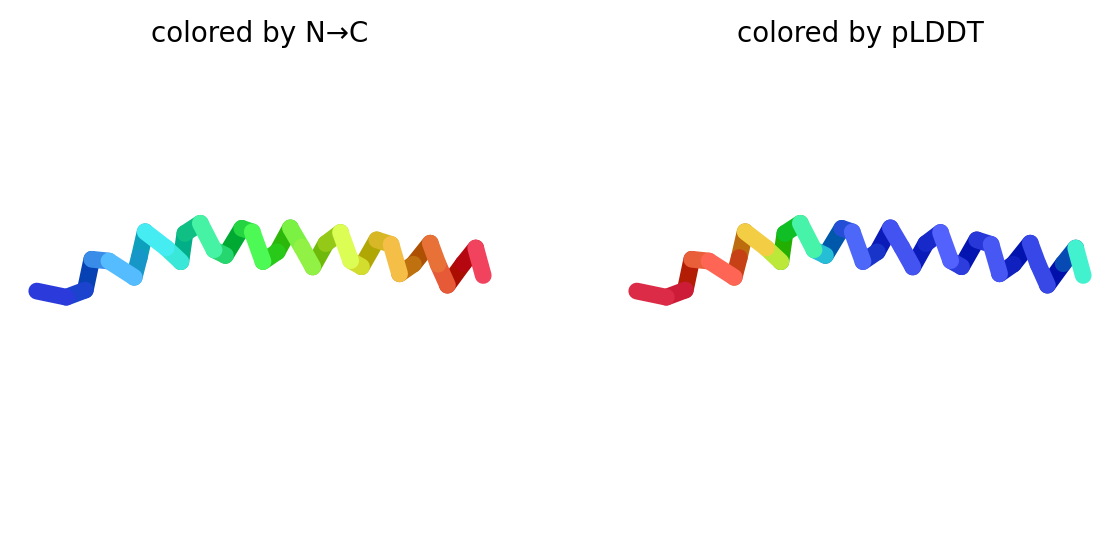

Supplement: lqaf178_Supplemental_Files [file lqaf178_supplemental_files.zip › DWORF/rank_5_model_2_ptm_seed_0.png]

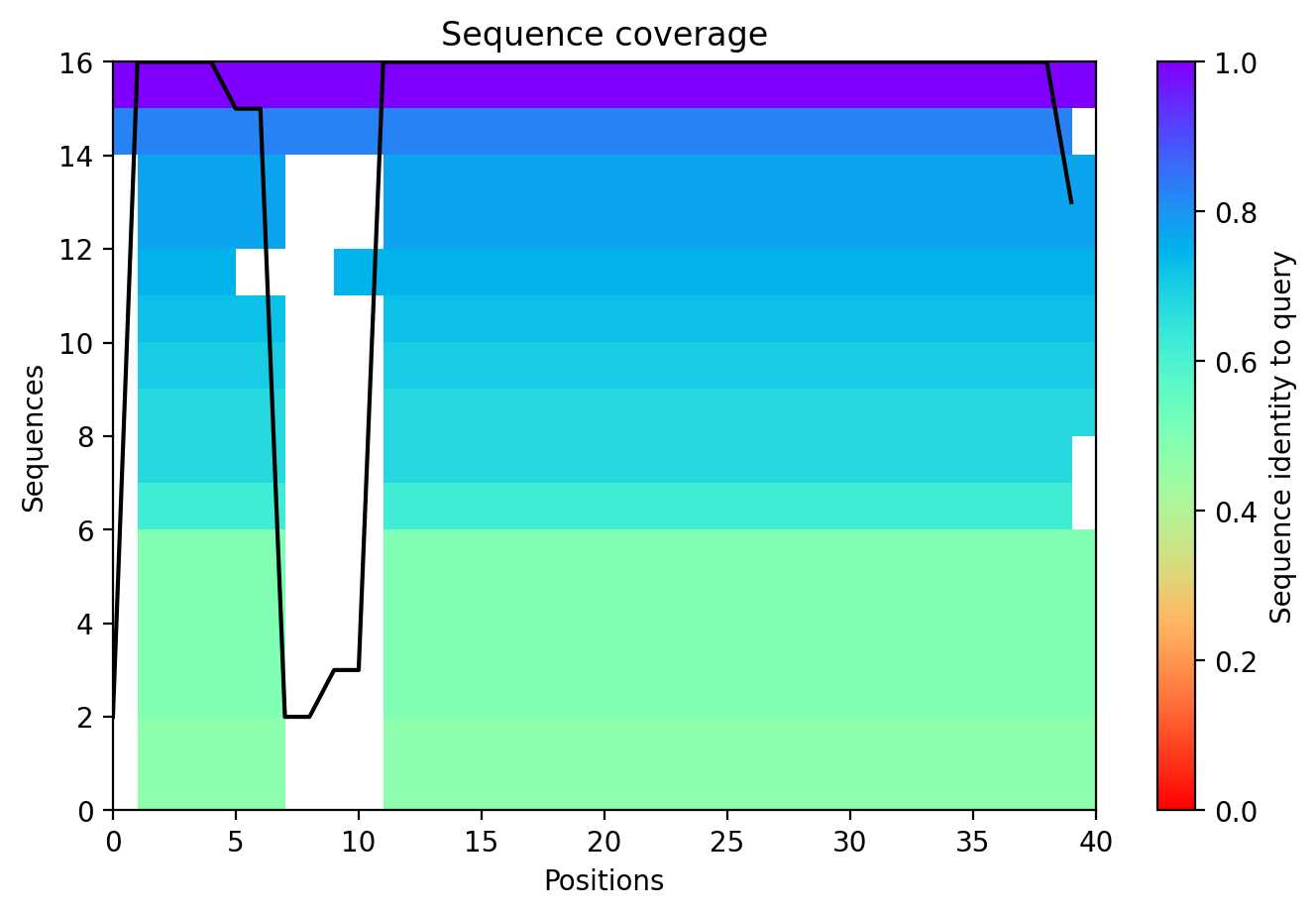

Supplement: lqaf178_Supplemental_Files [file lqaf178_supplemental_files.zip › F86JP/msa_coverage.png]

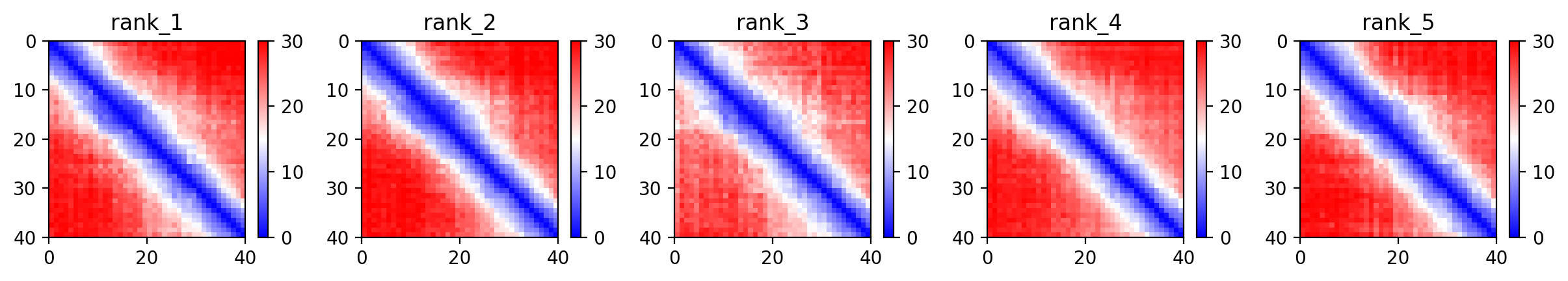

Supplement: lqaf178_Supplemental_Files [file lqaf178_supplemental_files.zip › F86JP/predicted_alignment_error.png]

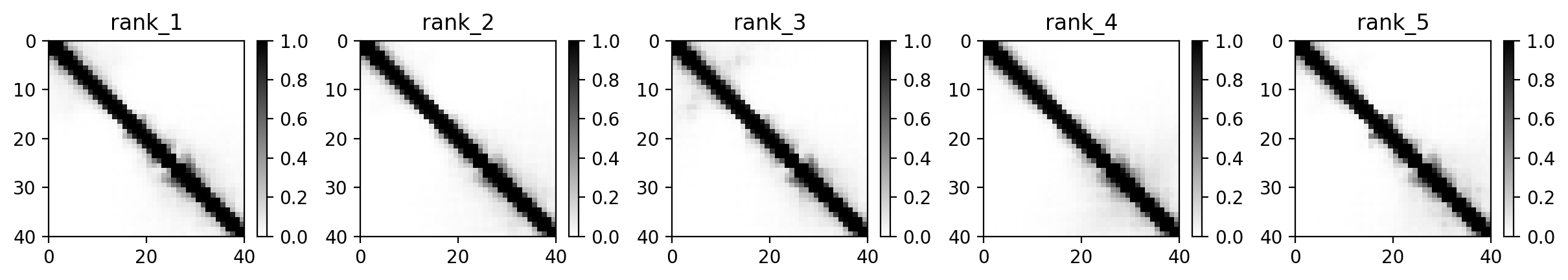

Supplement: lqaf178_Supplemental_Files [file lqaf178_supplemental_files.zip › F86JP/predicted_contacts.png]

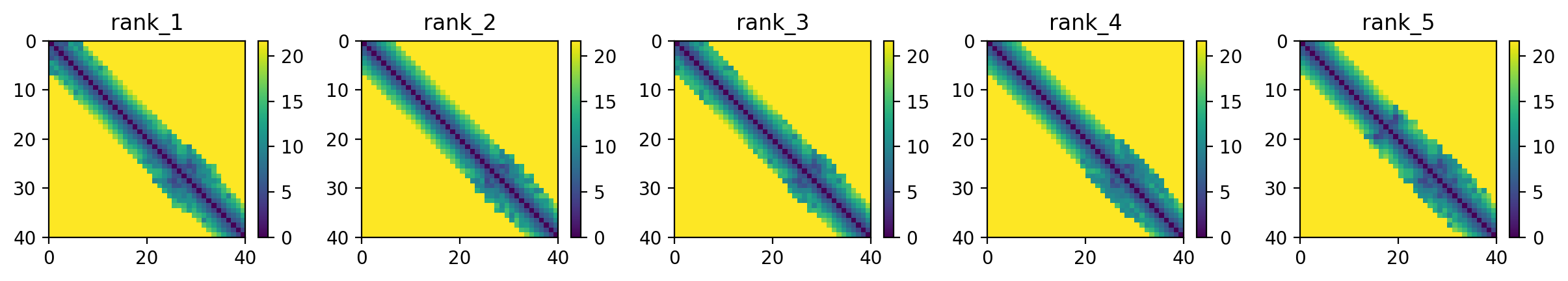

Supplement: lqaf178_Supplemental_Files [file lqaf178_supplemental_files.zip › F86JP/predicted_distogram.png]

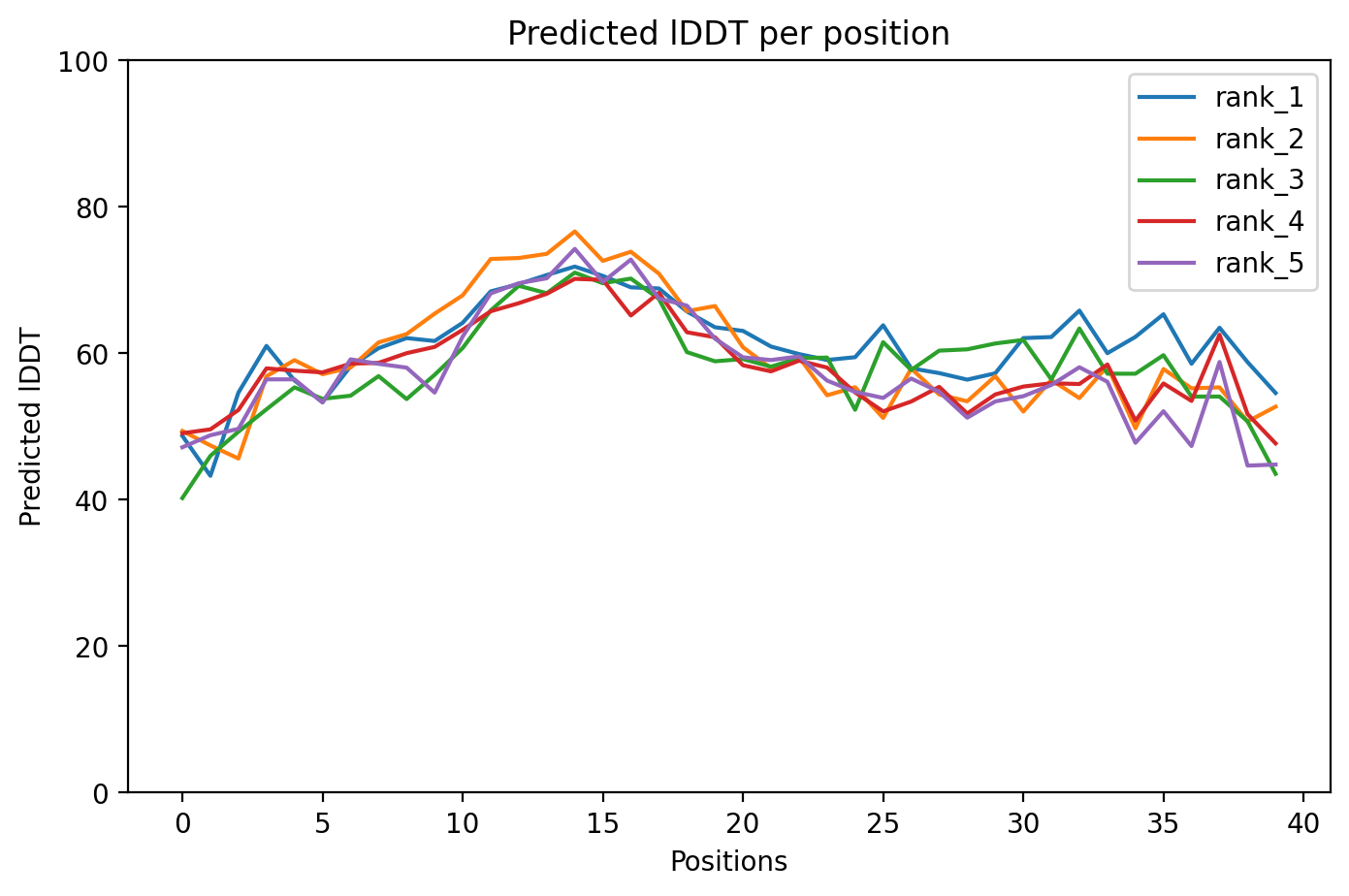

Supplement: lqaf178_Supplemental_Files [file lqaf178_supplemental_files.zip › F86JP/predicted_LDDT.png]

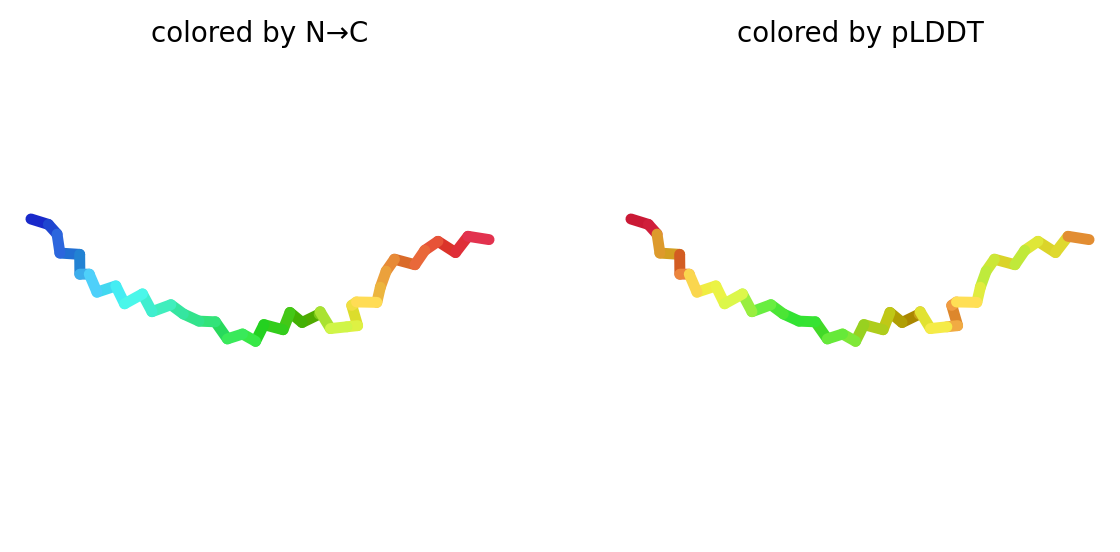

Supplement: lqaf178_Supplemental_Files [file lqaf178_supplemental_files.zip › F86JP/rank_1_model_2_ptm_seed_0.png]

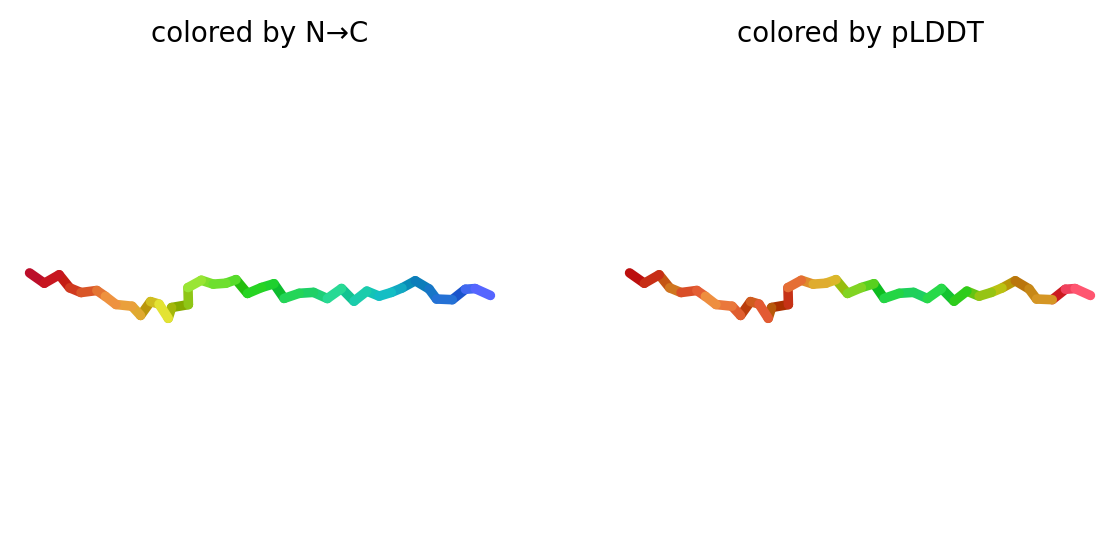

Supplement: lqaf178_Supplemental_Files [file lqaf178_supplemental_files.zip › F86JP/rank_2_model_4_ptm_seed_0.png]

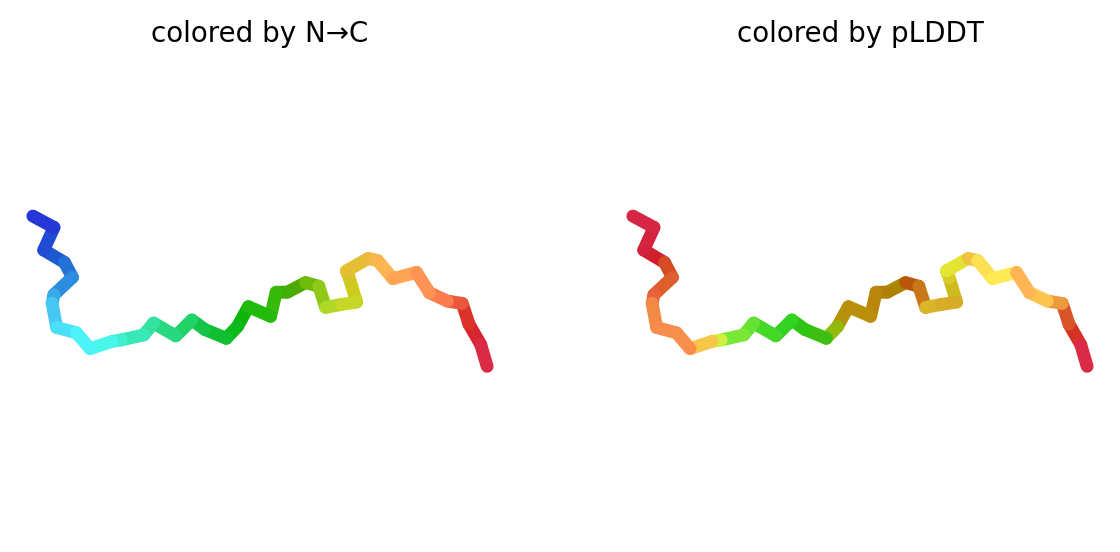

Supplement: lqaf178_Supplemental_Files [file lqaf178_supplemental_files.zip › F86JP/rank_3_model_1_ptm_seed_0.png]

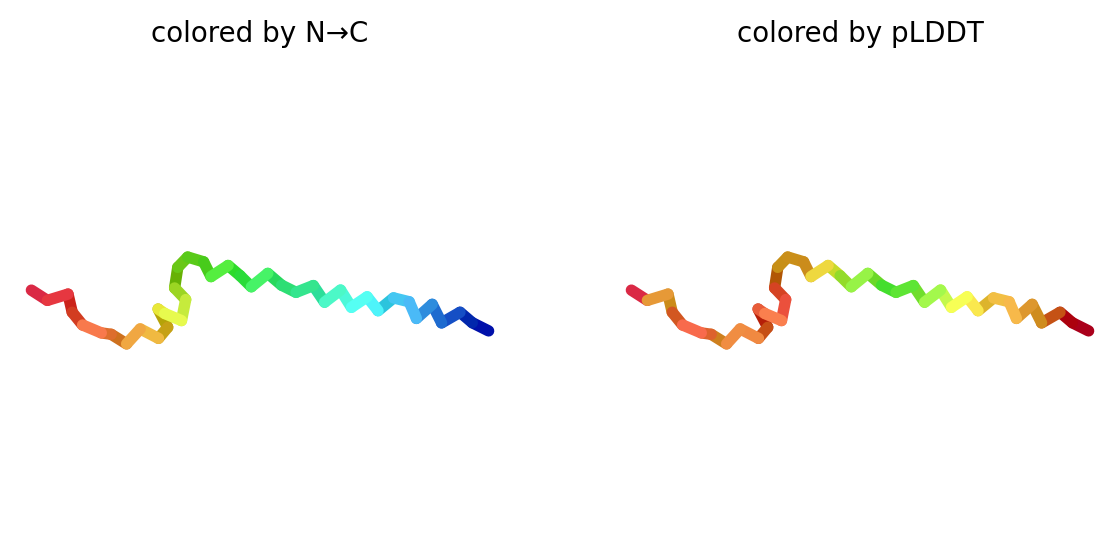

Supplement: lqaf178_Supplemental_Files [file lqaf178_supplemental_files.zip › F86JP/rank_4_model_3_ptm_seed_0.png]

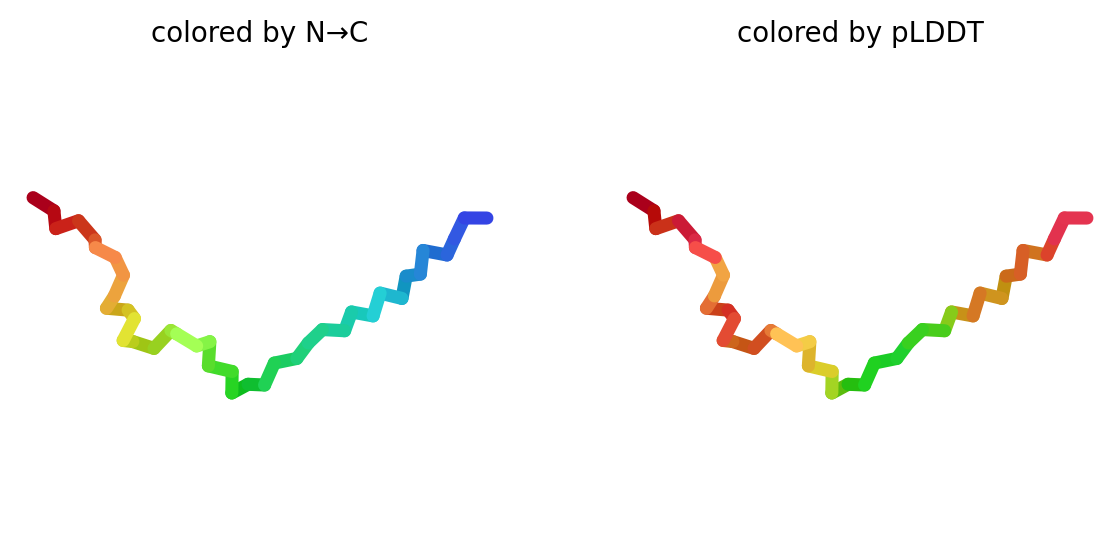

Supplement: lqaf178_Supplemental_Files [file lqaf178_supplemental_files.zip › F86JP/rank_5_model_5_ptm_seed_0.png]

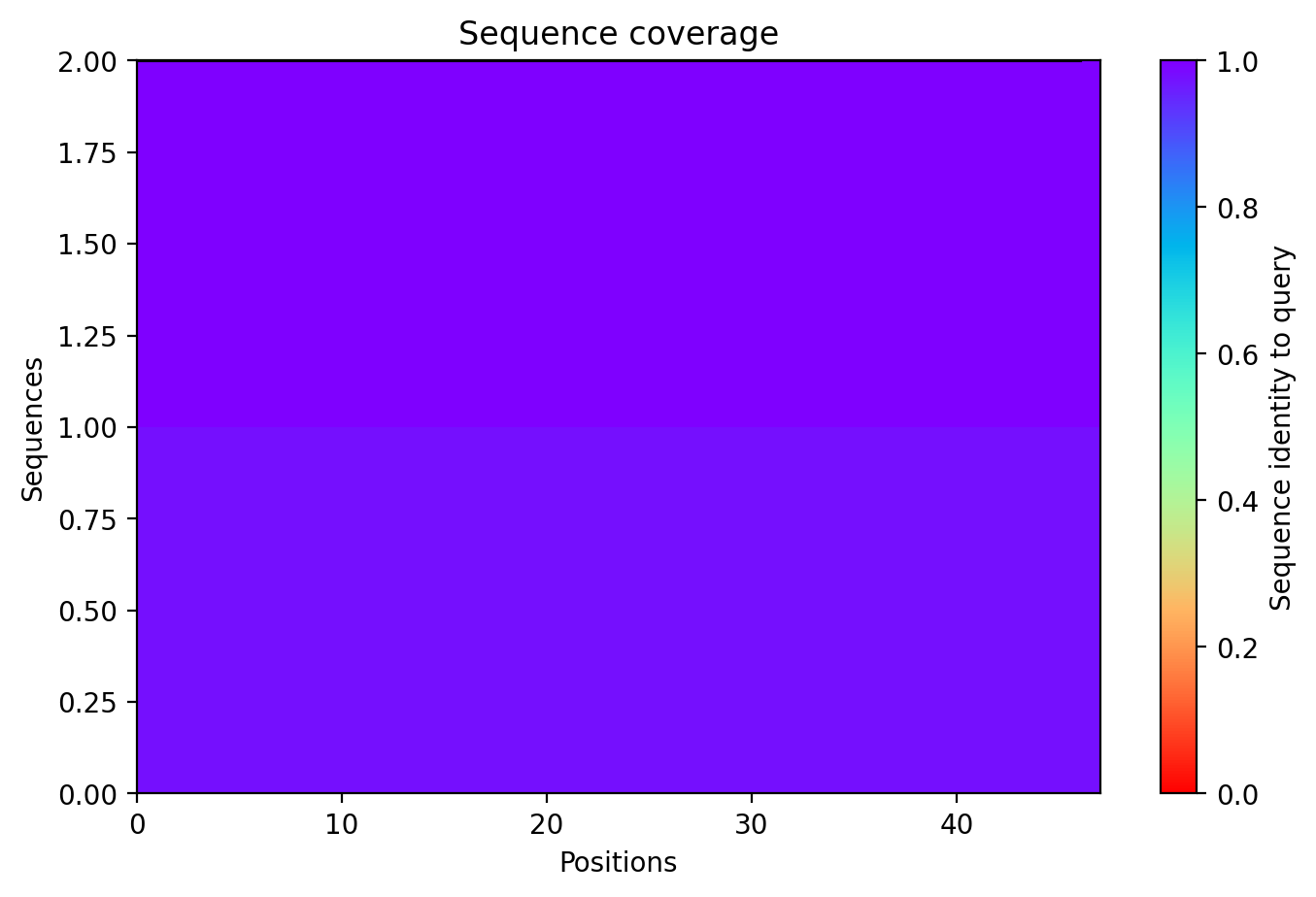

Supplement: lqaf178_Supplemental_Files [file lqaf178_supplemental_files.zip › FA66E/msa_coverage.png]

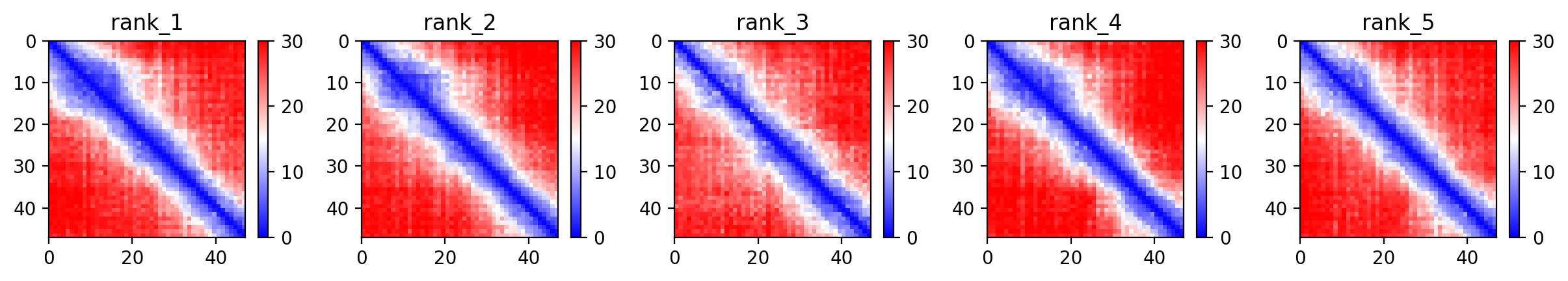

Supplement: lqaf178_Supplemental_Files [file lqaf178_supplemental_files.zip › FA66E/predicted_alignment_error.png]

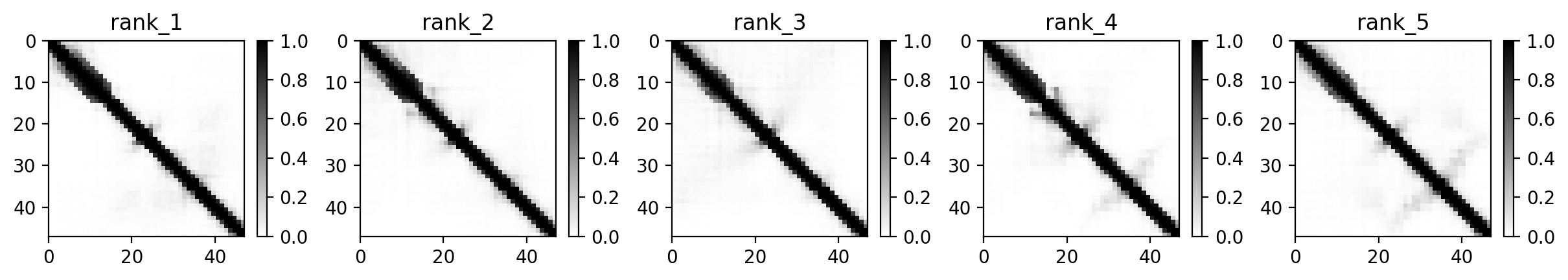

Supplement: lqaf178_Supplemental_Files [file lqaf178_supplemental_files.zip › FA66E/predicted_contacts.png]

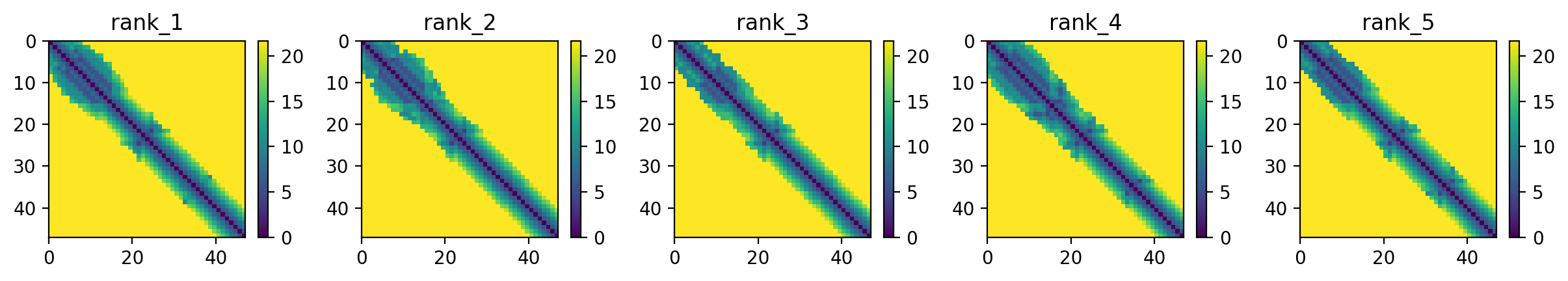

Supplement: lqaf178_Supplemental_Files [file lqaf178_supplemental_files.zip › FA66E/predicted_distogram.png]

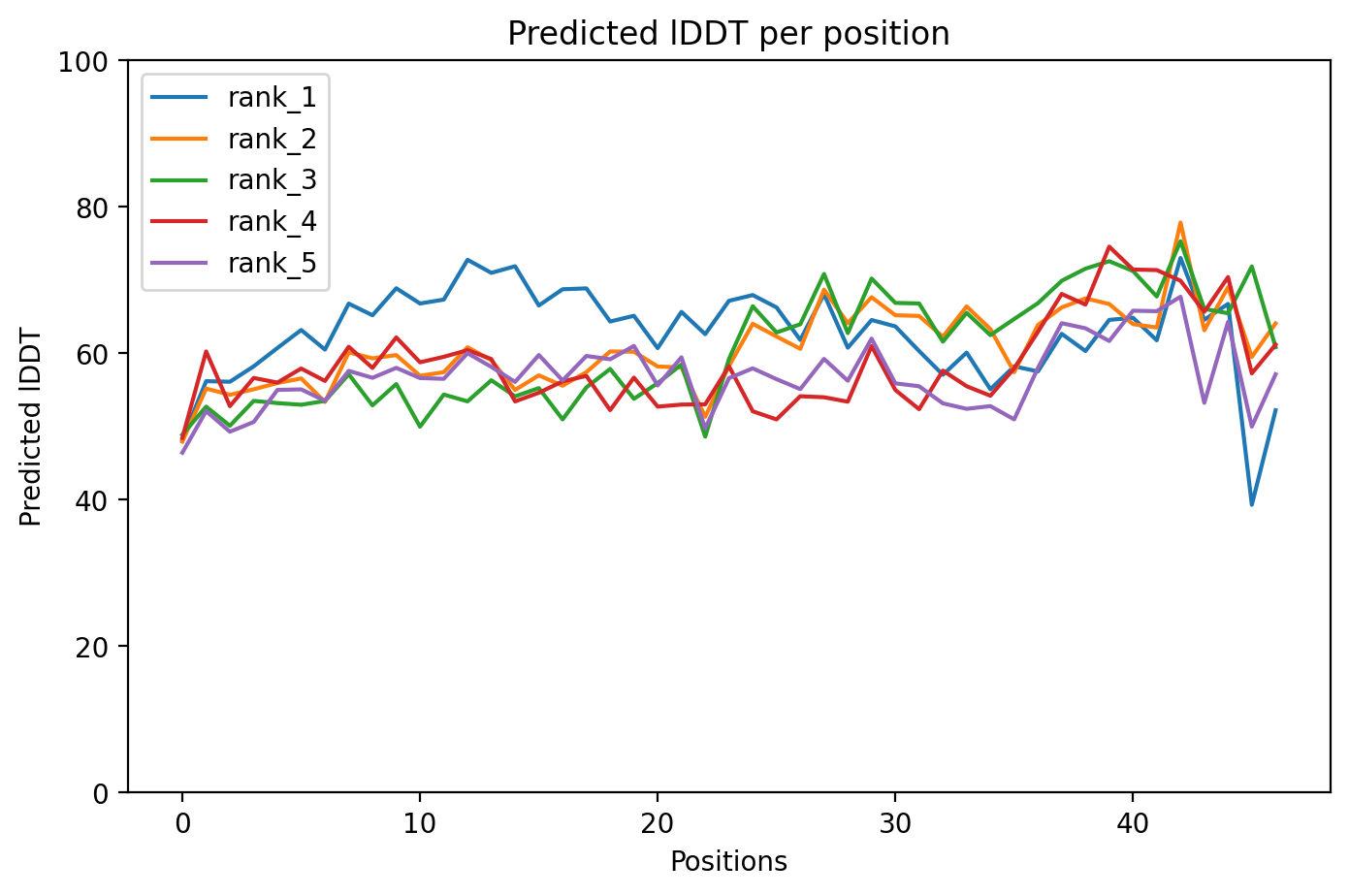

Supplement: lqaf178_Supplemental_Files [file lqaf178_supplemental_files.zip › FA66E/predicted_LDDT.png]

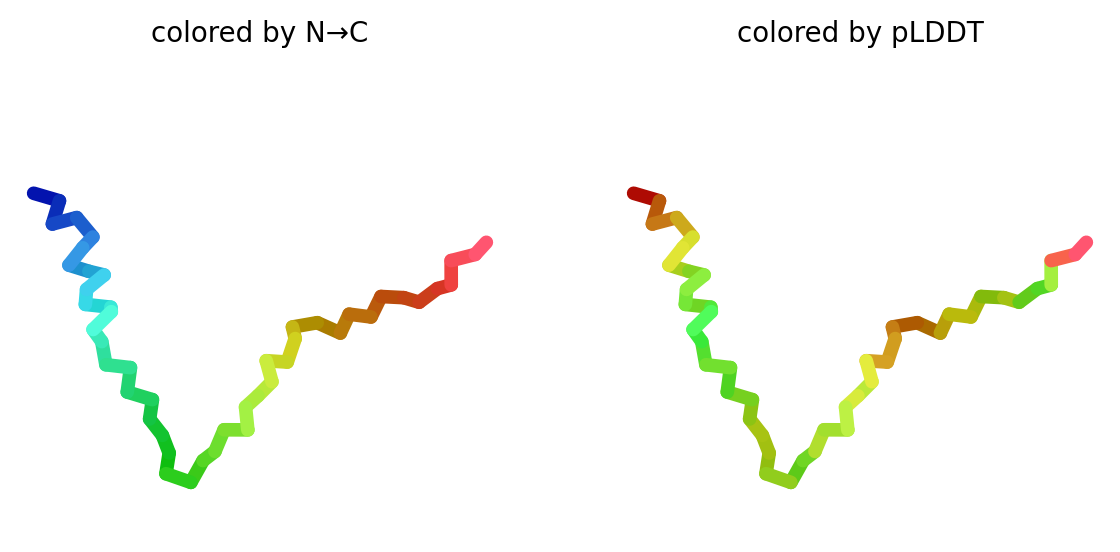

Supplement: lqaf178_Supplemental_Files [file lqaf178_supplemental_files.zip › FA66E/rank_1_model_3_ptm_seed_0.png]

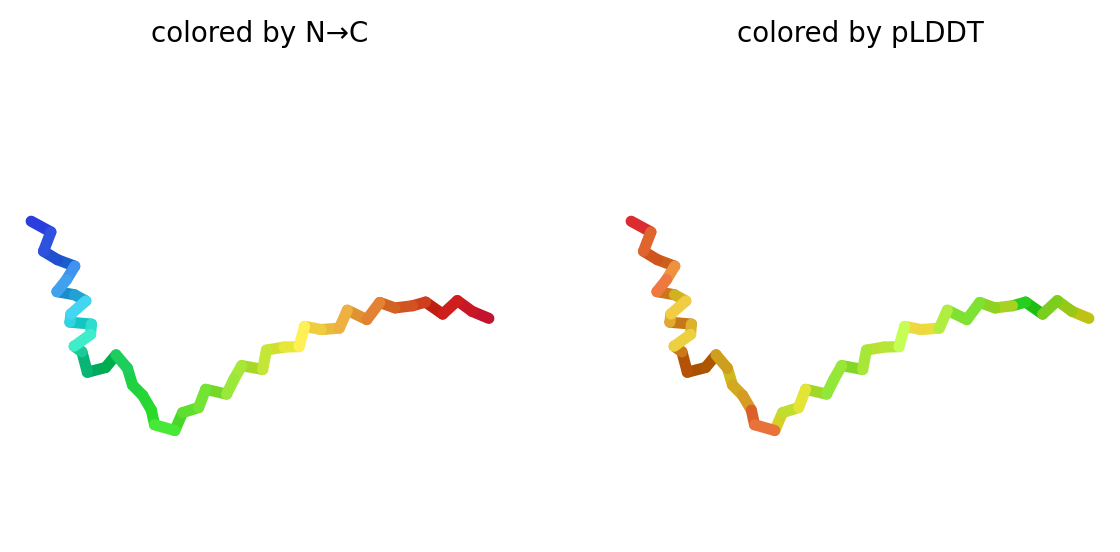

Supplement: lqaf178_Supplemental_Files [file lqaf178_supplemental_files.zip › FA66E/rank_2_model_2_ptm_seed_0.png]

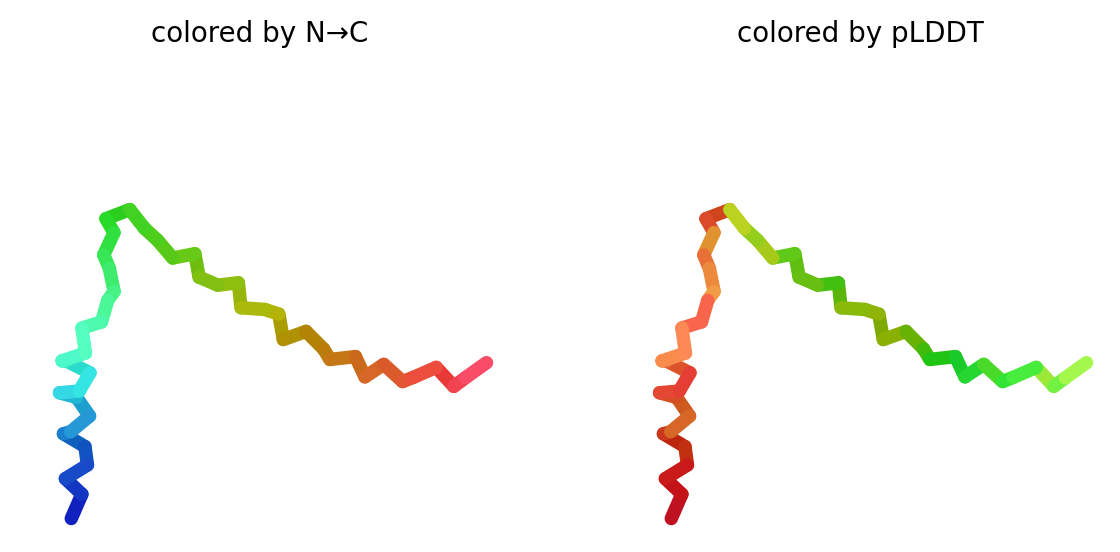

Supplement: lqaf178_Supplemental_Files [file lqaf178_supplemental_files.zip › FA66E/rank_3_model_1_ptm_seed_0.png]
